# Hierarchical Micro/Nano-Structured Biomaterials for Wound Healing

**DOI:** 10.34133/research.0968

**Published:** 2025-11-17

**Authors:** Minhui Lu, Zhiqiang Luo, Hanxu Chen, Yuanjin Zhao

**Affiliations:** ^1^ Department of Rheumatology and Immunology, Nanjing Drum Tower Hospital, School of Biological Science and Medical Engineering, Southeast University, Nanjing 210096, China.; ^2^ Wenzhou Institute, University of Chinese Academy of Sciences, Wenzhou, Zhejiang 325001, China.

## Abstract

Wounds have been an attractive research object in the biomedical field because of their high incidence and universality. Compared with traditional wound dressings such as gauze with a simple composition, hierarchical biomaterials with characteristics of micro/nano structure or micro/nano contents have been widely studied in view of the challenge in wound healing. In particular, a great deal of effort has been devoted to developing hierarchical biomaterials with specific curative features, such as antibacterial, antioxidant, and oxygen supply, which enable them to greatly promote wound healing. In this paper, the hierarchical biomaterials with a micro/nano structure for wound treatment are reviewed. After introducing the wound healing mechanism and key obstacles, the fabrication strategies and specific characteristics of the hierarchical biomaterials are discussed and summarized in detail. The emphasis is put on their application in different kinds of wounds. Finally, the future development prospects and direction of hierarchical biomaterials in the field of wound treatment are also addressed.

## Introduction

Wound refers to the damage or rupture of the surface layer of human skin or mucosa, frequently caused by collision, cutting, tearing, burn, and other external injury [1]. As an open disease, the healing process of wounds, including hemostasis, inflammation, proliferation, and remodeling, is easily hindered by external environmental factors such as bacterial infection [2,3]. In addition, the patient’s own systemic chronic diseases, such as diabetes, can also easily escalate the difficulty of wound healing, forming more refractory wounds, and even threatening life [4]. Traditional wound dressings employ gauze, bandage, spray, and others to isolate the wound from the external environment. Although they are sometimes supplemented with biomaterials like collagen or hyaluronic acid, they still do not participate in the physiological process of wound healing, which limits rapid and effective treatment of wounds [5]. However, with the development of life sciences, the mechanism of wound healing is increasingly understood. Many measures that are conducive to wound healing have been proposed, such as the removal of harmful bacteria, the reduction of oxidative stress levels, and adequate oxygen supply [6]. Therefore, these more refined requirements demand new features of the materials, and more functional wound dressings are expected.

Hierarchical micro/nano-structured biomaterials are kinds of macroscale biomaterials with micro/nano-scale structures or carrying micro/nano-size components, such as common porous structures or encapsulated nanoparticles [7,8]. The skeleton components of hierarchical micro/nano-structured biomaterials are diverse, which can be composed of materials derived from natural biological matrix and their derivatives [9]. Besides, the biomaterials can be further modified based on special fabrication processes. Through the selection of raw materials, hierarchical micro/nano-structured biomaterials usually have good biocompatibility [10]. Moreover, benefitting from both the micro/nano structure and material properties, the resulting hierarchical biomaterials can be endowed with a variety of curative abilities, such as controlled drug delivery, antibacterial, adhesion, antioxidant, and sensing [11,12]. General biomaterial wound dressings, such as gelatin, pectin, carboxymethylcellulose, and alginate, only act as temporary skin substitutes, which absorb exudate and become a barrier to protect the wound from external bacteria and others. They are less involved in the wound healing process, or promote wound healing. In contrast, such diversiform functional hierarchical biomaterials can help to fit the different types of wounds and accurately regulate the physiological process of wound healing, ultimately achieving better wound healing performance than traditional dressings and general biomaterial wound dressings. Hence, hierarchical biomaterials play an important role in recent wound healing research.

**Fig. 1.**
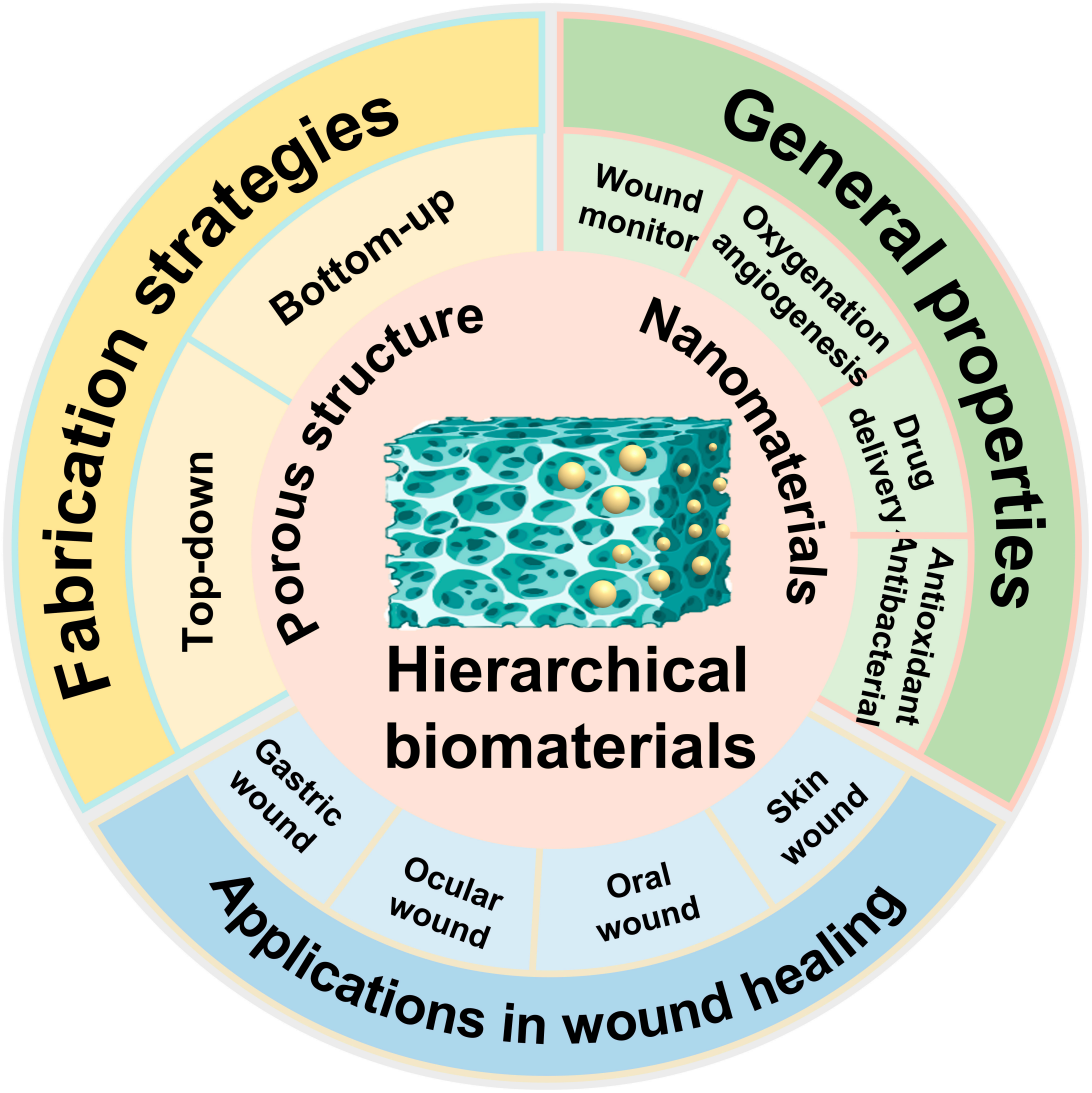
Hierarchical micro/nano-structured biomaterials for wound healing.

In this review, we detail the superior properties of hierarchical micro/nano-structured biomaterials in promoting wound healing (Fig. 1). Specifically, we start with some basic concepts to gain a clear understanding of the physiological process of wound healing represented by skin, followed by the key influencing factors in wound healing. A variety of preparation methods of hierarchical biomaterials based on the basic strategy of top-down or bottom-up are then introduced. Subsequently, the topic focuses on the curative properties of hierarchical biomaterials in wound healing, and their application in wounds on different organs, as well as the unique characteristics required are discussed. Finally, the possible developmental direction of hierarchical micro/nano-structured biomaterials for wound treatment is also analyzed. We look forward to this review, which will help readers to further broaden their understanding of hierarchical biomaterials and guide efforts to promote clinical advances in the treatment of wound diseases.

## Fundamental Mechanism of Wound Healing

Similar to the development of other diseases, refractory wound healing has a unique physiological process and is also affected by internal and external physiological factors. Mastering the mechanism of wound healing is the premise of symptomatic treatment. In this section, we will introduce the typical wound healing process and key threats to hamper healing (e.g., bacterial infection and diabetes) in detail to understand the current challenges in wound healing.

### Process of wound healing

Skin is the most common subject of wound healing studies. Understanding the normal structure of the skin is a prerequisite for discussing the process of wound healing. It is a truism that the main components of skin are the epidermis and dermis. The epidermis is the outermost layer of human tissue, containing the keratinocytes. Keratinocytes in the basal layer have the strongest proliferation ability and is the main force of wound regeneration and repair. The dermis consists of connective tissues and is rich in fibroblasts that provide toughness and elasticity to the skin. At the same time, capillaries, nerves, and other structures mainly exist in the dermis, which is the source of wound bleeding and pain [13,14].

**Fig. 2.**
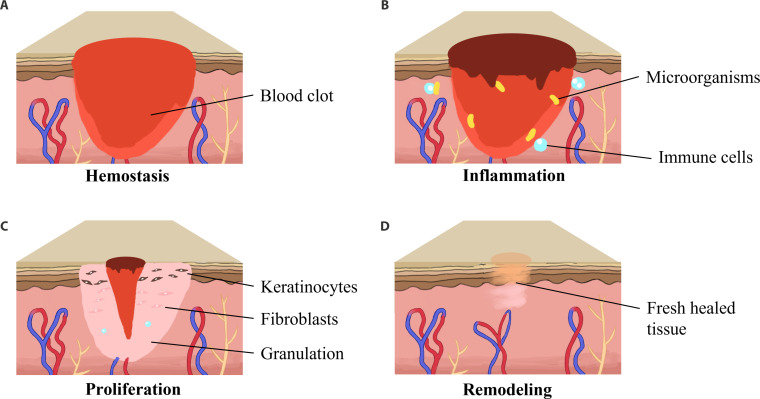
Schematic diagram of the wound healing process including (A) hemostasis, (B) inflammation, (C) proliferation, and (D) remodeling.

Once the skin is damaged, a highly regulated and dynamic repair process begins immediately. In general, the healing process of both acute and chronic wounds is classified into 4 overlapped stages: hemostasis, inflammation, proliferation, and remodeling (Fig. 2) [15–17]. Hemostasis occurs at the moment of injury. Regulated by neural reflex mechanisms, injured blood vessels rapidly constrict to limit blood loss. Meanwhile, platelets and clotting factors form a blood clot. In addition to preventing bacterial invasion, the blood clot also acts as a scaffold for later cell migration and a storage of growth factors, affecting the subsequent healing stage [18]. The inflammatory response period after trauma mainly occurs from the immediate post-injury to 48 h, which is the primary activation of the innate immune system to eliminate microorganisms and damaged endogenous tissues at the wound site. The immune cells involved in this process also secreted different growth factors and chemokines, attracting more cells to promote the tissue repair of the wound, thus directing the healing process into the proliferation stage [19]. On about the third day after injury, as the inflammatory response subsides and the tissue repair cells gradually proliferate, the wound repair ushers in a proliferative period. The proliferation stage is characterized by neovascularization, granulation tissue formation, and completion of epithelialization [20]. Granulation tissue is a pale pink tissue composed of fibroblasts and keratinocytes, containing immune cells and new capillaries that fill the gap in the skin caused by trauma. During the generation of granulation tissue, basal layer cells near the wound surface also proliferated and migrated to generate fresh epithelial cells [21]. On the other hand, the tissue characteristics of the proliferative stage are also reflected in the aspect of collagen deposition. The production of type III collagen is higher than its decomposition at this stage [21]. Remodeling is the process of transforming regenerated granulation tissue into an integrated, closed wound. In this process, production and decomposition of collagen become balanced, where type III is gradually replaced by type I. Besides, the collagen arranges in orderliness, which increases the intensity of the new tissue [22]. Some capillaries transform into arterioles or veins, and the skin appendages regenerate. The matrix constructed in the previous tissue diverts to form functional cutaneous tissue or semi-/nonfunctional scar tissue [15]. Scar formation is one of the typical outcomes of wound healing in soft tissue. For wounds with few defects, neatly aligned, and noninfected wounds, healing can be completed within 2 to 3 weeks, while they can be delayed to 4 to 5 weeks to form scars when severely infected, and the scars are widespread, obstructing the view and even affecting function.

### Key factors in wound healing

The prolongation or obstruction of the wound healing process may cause a chronic wound. There is no doubt that wound healing is affected by many factors, such as age, gender, and other individual differences. Besides, there are some key factors that can also seriously affect wound healing, such as bacterial infection and chronic diseases (typically diabetes).

There are unique microbiomes on the skin to keep its microenvironment balanced. Some of them can act as a part of the skin barrier to protect the skin from pathogens. For example, the presence of *Staphylococcus epidermidis* can promote the integrity of the barrier and limit the colonization of pathogens [23]. In the process of wound healing, *S. epidermidis* can even reduce the inflammation response, which is conducive to the transition of the wound from the inflammatory stage to the proliferative stage, and participates in the formation of epithelialization and granulation tissue during proliferation [17]. However, when the homeostasis of the microbiome is disrupted, the colonization of harmful bacteria will have an adverse effect on wound healing. For instance, an excess amount of *Staphylococcus aureus* leads to wound infection. *S. aureus* would promote inflammation, increase the content of reactive oxygen species (ROS) and other substances, and destroy the permeability of wound tissue to spread bacteria, which harms normal cells in the wound site and hinders wound healing [24]. In addition, biofilm produced by bacterial infection is also one of the reasons why more than 60% of chronic wounds are difficult to heal, for it resists the phagocytosis from host immune response and restricts the diffusion of drugs and antibodies [25]. In short, the microbiota in the wound plays a dual role in wound healing, and maintaining the microbiota homeostasis has significance in wound healing.

Diabetes is one of the most typical chronic diseases that greatly affects wound healing, which is a metabolic disease with the marked characteristic of hyperglycemia. It affects various systems and organs of the whole body, obstructs immune function, and causes a variety of complications in patients, where chronic wounds are one of the most common [26]. Diabetes influences multiple stages of wound healing [27]. Firstly, in the inflammatory period after hemostasis, the chemotaxis and phagocytosis of leukocytes are weakened due to the dysregulation of the inflammatory response in diabetic patients [28]. In particular, neutrophil cells and macrophages remain in the wound area, forming an environment with rich inflammatory factors and ROS, where the tissues would be damaged, and subsequent cell proliferation is prevented. Bacterial fragments accumulating without cleaning up in a timely manner reduce the expression of growth factors such as vascular endothelial growth factor, which hinders the transition of the wound from the inflammatory stage to the regenerative stage [29]. Secondly, in the regenerative stage, cell proliferation and differentiation are constrained by the hyperglycemia and impaired vascular function in diabetic patients [30]. In addition, diabetes can also cause oxidative stress and metabolic disorders in cells, making cells insufficient in energy. Finally, when entering the remodeling stage, the synthesis and deposition capacity of collagen in diabetic patients are diminished, which impedes the repair of wound tissue structure [31]. During the long-term process of chronic diabetic wounds, a warm and humid environment is more attractive to the colonization of harmful bacteria such as *S. aureus*, further resulting in an increased risk of bacterial infection.

Depending on the characteristics of different stages during wound healing and the manifestations of refractory wounds, researchers can intervene or monitor relevant physiological indicators at appropriate times, such as applying antibacterial measures and removing ROS during the inflammatory phase, and providing oxygen or promoting angiogenesis during the proliferation phase.

## Fabrications of Hierarchical Biomaterials

The fabrication of hierarchical biomaterials varies depending on the material properties and intended application. Hierarchical biomaterials are in diverse forms, including microspheres, fibers, films, and microneedles, to meet the demands of different applications. Here, we list the common preparation methods, including bottom-up strategies represented by self-assembly, phase separation, and microfluidics, and top-down strategies represented by 3-dimensional (3D) printing and micromanufacturing.

### Bottom-up strategies

#### Self-assembly

**Fig. 3.**
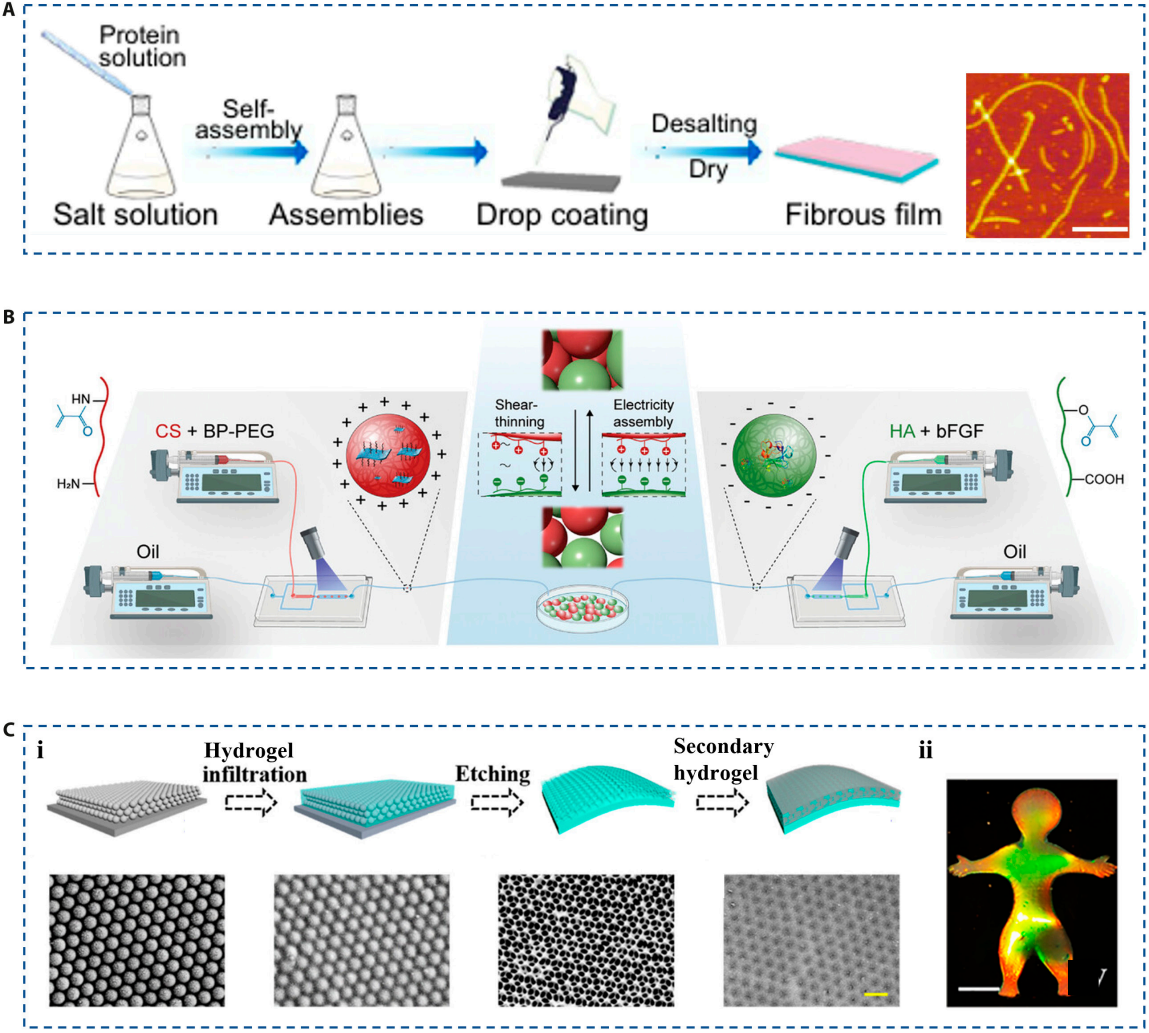
Fabrication of hierarchical biomaterials by self-assembly. (A) The preparation and characterization of hierarchical structured films containing fibrous chains of insect proteins [34]. Copyright 2023, American Chemical Society. (B) Preparation of microspheres containing positive and negative charges and a schematic representation of their self-assembly [35]. Copyright 2023, Wiley-VCH GmbH. (C) (i) Schematic diagram of the preparation and characterization of the reverse opal film, and (ii) the image of the reverse opal film with vivid structural color [40]. Copyright 2023, American Chemical Society.

Self-assembly refers to the formation of ordered structures mediated by chemical or physical interactions [32]. The molecular self-assembly is a common method for the preparation of hierarchically structural materials, which can realize the customization of functions and the control of morphology and size on the nanoscale [33]. Typically, Gong et al. [34] investigated an even beaded nanofibril film formed by controllable self-assembly of insect cuticle protein. The protein’s assembly was triggered by an increase in salt concentration and could also be caused by a higher pH value. Then, the author prepared the fiber film on the hydrophilic substrate by the drop coating method and proved its potential application in the antibacterial treatment of wounds (Fig. 3A). In addition to the molecules’ self-assembly into products on the nanoscale, researchers have proposed a strategy for the self-assembly of microspheres into hydrogel scaffolds [35]. In particular, the positively charged microspheres of chitosan methacryloyl could combine with the negatively charged microspheres of hyaluronic acid methacryloyl through electrostatic interaction (Fig. 3B). This study provided a convenient strategy for the design and fabrication of novel hydrogel-based hierarchical biomaterials with free-editing and combination capabilities. Colloidal crystals are a kind of ordered structure material formed by the self-assembly of colloidal nanoparticles. Although the colloidal crystals have a photonic band-gap characteristic because of the orderly arrangement of nanoparticles, shown as bright structural colors, their application in the biomedical field is still limited by their dense, rigid structure. Therefore, hydrogel inverse opals replicated from colloidal crystal templates are developed. They possess similar structural and optical properties to colloid crystals. Moreover, inverse opal materials have more striking features than colloidal crystals, such as open and interconnected porous structures, high specific surface area, and multifunctional properties, which are derived from hydrogel properties (Fig. 3C) [36–39]. For instance, Wang et al. [40] proposed an inverse opal patch scaffolded with ionic hydrogel, which is conductive, freeze-resistant, and structurally colored. When combined with the microfluidic technology, silica colloidal crystals in the form of microspheres can be fabricated in large quantities. For example, Chen et al. [41] utilized chitosan as a scaffold hydrogel to fabricate inverse opal microspheres, which could be further sprayed on the wound site to deliver drugs. Meanwhile, Wang et al. [42] proposed self-healing inverse opal microparticles by using a dynamic hydrogel that could adhere together.

#### Phase separation

**Fig. 4.**
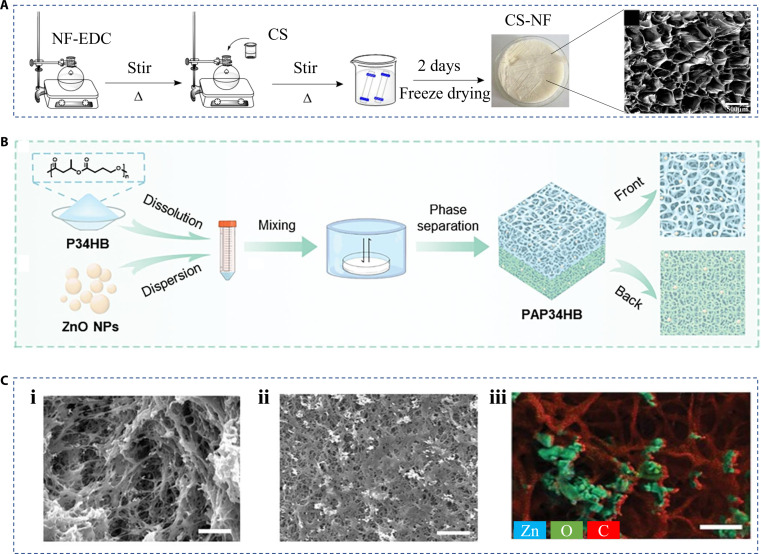
Fabrication of hierarchical biomaterials by phase separation. (A) A porous sponge prepared by freeze-drying [44]. Copyright 2022, Elsevier B.V. All rights reserved. (B) A porous polycrystalline silicate scaffold prepared by solvent exchange phase separation [46]. (C) The different pore structures of polycrystalline silicate scaffold on (i) front and (ii) back. (iii) Energy-dispersive x-ray elemental analysis of P34HB-based scaffolds [46]. Copyright 2022, Elsevier B.V. All rights reserved.

Phase separation is a physicochemical concept in which, under certain conditions, a binary or multivariate mixture will separate into different phases with different physicochemical properties. The pores in hierarchical biomaterials could be obtained directly by phase separation [43]. For example, Cheng et al. [44] prepared a norfloxacin-grafted chitosan antimicrobial sponge with a porous structure by freezing-induced phase separation, which was also known as the freezing drying strategy (Fig. 4A). Apart from freezing, a rapid thermal annealing way to activate phase separation was proposed by Pagaduan et al. [45]. They successfully fabricated controllable porous carbon network films and investigated their potential application in wound healing. Specifically, the material was heated to 300 °C at a rate of 10 °C/s, lasting 10 min to achieve phase separation and cross-linking. It was then heated at the same rate to 800 °C in a nitrogen atmosphere lasting 1 min, during which time the pores formed. Taking advantage of the difference in solubility of solvents in different solutions is also one of the ways of phase separation. Chen et al. [46] developed a porous poly-droxyalkanoates scaffold with ZnO nanoparticles via solvent exchange and phase separation (Fig. 4B). When the chloroform, initial solvent of poly-droxyalkanoates, was gradually replaced by a mass of alcohol, the scaffold was left at the bottom of the container. In particular, the scaffold had asymmetrical porosity where the front was made up of porous spongy parts and the back consisted of low-porosity dense layers (Fig. 4C). The formation of these structures can be attributed to nucleation and accelerated crystal growth.

#### Microfluidics

**Fig. 5.**
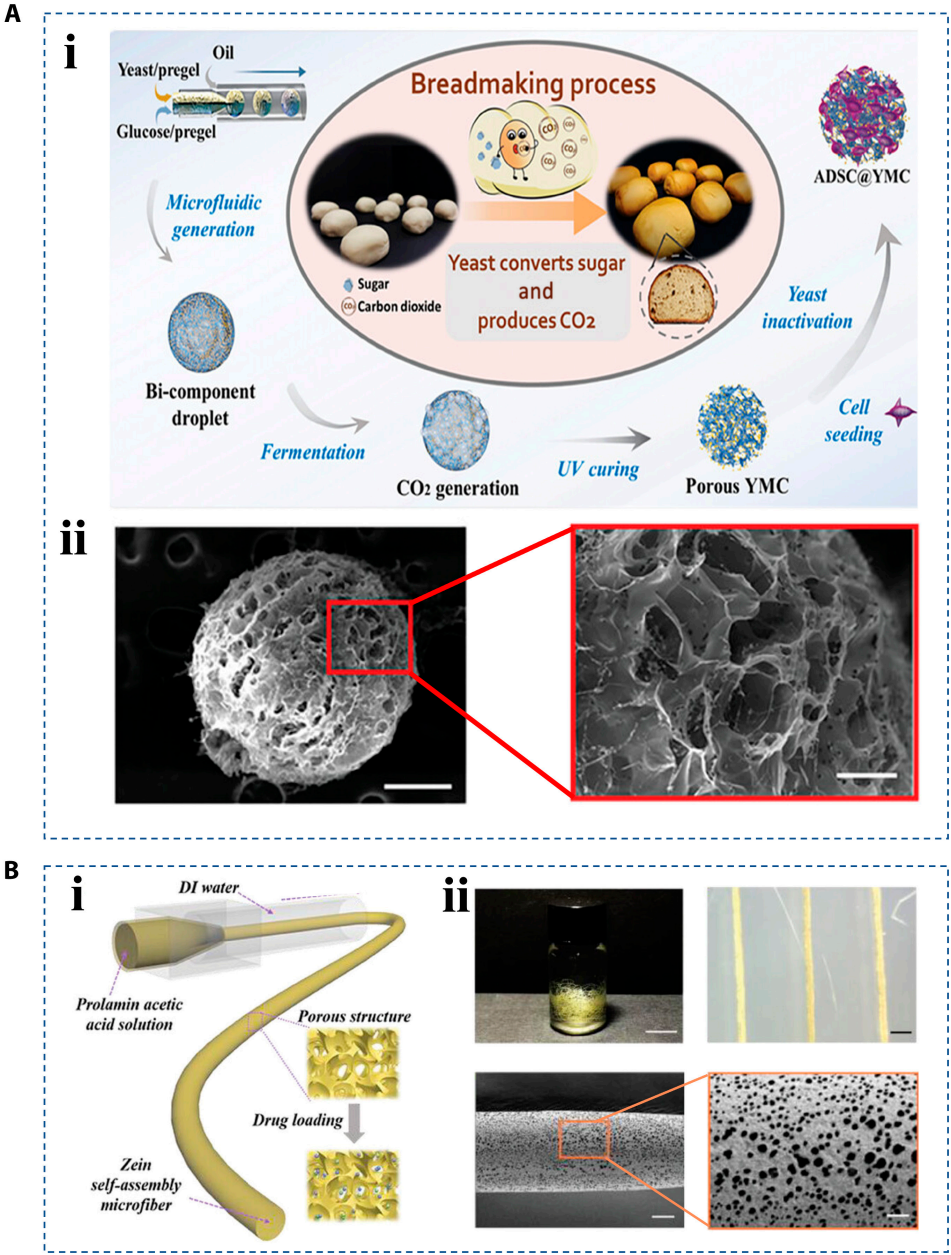
Fabrication of hierarchical biomaterials by microfluidics. (A) (i) The preparation schematic diagram of porous microspheres by droplet microfluidics technology inspired by bread making. (ii) Characterization of these porous microspheres [55]. Copyright 2023, Wiley-VCH GmbH. (B) (i) The preparation schematic diagram of porous microfibers. (ii) Optical images and SEM images of these porous microfibers [58]. Copyright 2023, Wiley-VCH GmbH.

Microfluidics technology enables the precise handling and control of minute volumes of fluids using microchannels and has been widely applied in biomedicine and other fields [47,48]. Using microfluidics, researchers can produce a wide variety of hierarchical microparticles and microfibers [49–51]. After generation of monodisperse droplets from microfluidics, they remain spherical due to surface tension, acting as droplet templates [52,53]. In the simplest case, homogeneous polymer particles, composed of either polymer chains or interlinked networks, would be synthesized through curing those resultant droplets. Based on this principle, through the design of microfluidic channels and fluid components, microspheres containing a porous structure, hierarchical core–shell structure capsules, and vesicle structure liposomes could be fabricated [54]. For instance, Wu et al. [55] combined the principle of bread making and microfluidics to prepare porous microsphere carriers. Specifically, a dual-channel microfluidic device was first utilized to create a Janus hydrogel precursor droplet, with yeast in one hemisphere and glucose in the other. These Janus droplets were then introduced into the fermentation tunnel, where yeast and glucose combine to produce gas. Subsequently, the droplets were polymerized by ultraviolet light, the porosity of which can be adjusted by adjusting the fermentation time (Fig. 5A). The preparation of microfibers reduces the effect of fluid shear forces relative to microparticles, so that the fluid can continuously form a fibrous structure [56]. Hydrogel microfibers typically have properties of high moisture content, ease of weaving, and satisfactory physical strength and flexibility, which allow them to play a crucial role in keeping the wound environment properly hydrated despite the water absorption by skin under strong stress [57]. Wang et al. [58] employed a protein acetic acid solution as a matching deionized solution to fabricate microfibers. Through modulating the protein concentration and the flow rate parameters in the microfluidic process, precise tailoring of the microfibers’ pore architecture, morphological features, and diameter dimensions became achievable (Fig. 5B). Due to its porous nature, the resultant microfibers can be used as flexible delivery systems for bioactive substances such as bacitracin and vascular endothelial growth factor.

### Top-down strategies

#### 3D printing

**Fig. 6.**
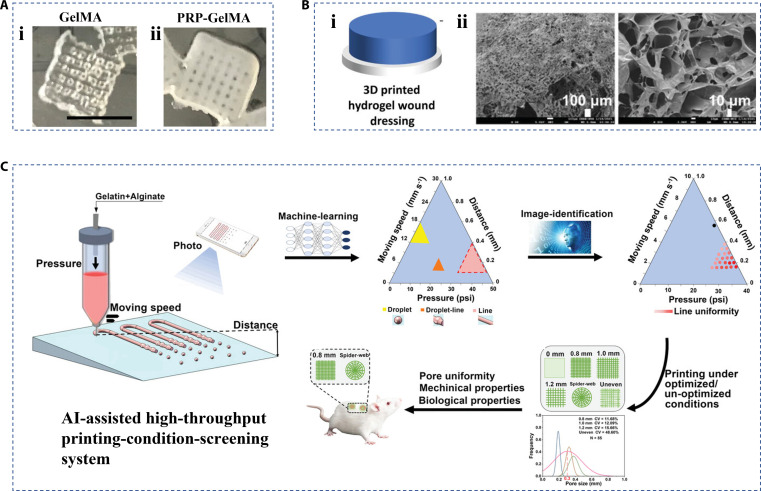
Fabrication of hierarchical biomaterials by 3D printing. (A) Optical images of (i) GelMA and (ii) PRP-GelMA hydrogel scaffolds [62]. Copyright 2021, Wiley-VCH GmbH. (B) (i) The preparation schematic diagram and (ii) characterization of porous chitosan methacrylate hydrogel scaffolds [63]. Copyright 2021, Wiley-VCH GmbH. (C) The schematic diagram of an artificial intelligence-assisted high-throughput printing-condition-screening system for fabricating hierarchical biomaterials [65]. Copyright 2022, Wiley-VCH GmbH.

3D printing is an emerging fabrication strategy to fabricate the controllable hierarchical porous structure by printing samples layer by layer using curable materials based on design drawings [59–61]. Benefitting from the advantage of producing complex structures of various shapes and sizes, a variety of biomaterials and their derivatives have been developed as bio-ink for 3D printing in the field of tissue engineering. For example, gelatin methacrylate (GelMA) was obtained by adding methacrylate groups to the amino groups in gelatin. The introduced methacrylate groups allow for polymerization upon exposure to the high-energy excitation ray with the existence of a photo-initiator. During this reaction, double bonds are broken, followed by new covalent bonds between gelatin being created. For instance, Ibanez et al. [62] developed one kind of bio-ink based on the GelMA. The platelet-rich plasma (PRP) was combined with GelMA molecules, which were then introduced to the 3D-printed framework and provided the enhancement in angiogenesis (Fig. 6A). In addition to GelMA, the chitosan, a biodegradable hydrogel, can also be used as a bio-ink through a similar preprocessing. Teoh et al. [63] demonstrated the effectiveness of chitosan methacrylate in 3D printing. The porous microstructure from chitosan methacrylate at low concentration provided space for drug upload while allowing exudate and drug transformation between the dressing and the wound site (Fig. 6B). When compatible with drugs, the personalized wound dressing also has multiple functions, such as local analgesia and anti-infection. Coincidentally, carboxymethyl chitosan, another derivative of chitosan, was 3D printed into porous hydrogel scaffolds with oxidized alginate and assembled with a hydrophobic electrospinning layer grafted with catechol to form a double-layer skin substitute [64]. Although 3D printing has the ability to manufacture complex structures, the uniformity of its scaffold preparation depends on the optimization of printing parameters, which, in turn, depends on prior experience. Therefore, traditional 3D printing technology is time-consuming and has a high technical threshold. In contrast, Chen et al. [65] presented the artificial intelligence-assisted high-throughput printing-condition-screening system to overcome these disadvantages (Fig. 6C). Based on the system, the researchers were able to screen the optimal printing conditions with high throughput. The resultant alginate-gelatin wound dressing from 3D printing indicated that the uniformity of the scaffold was improved, and it had good mechanical and biological properties in wound healing. This unique system is expected to simplify the manufacturing process of 3D printing technology in tissue engineering.

#### Micromanufacturing

**Fig. 7.**
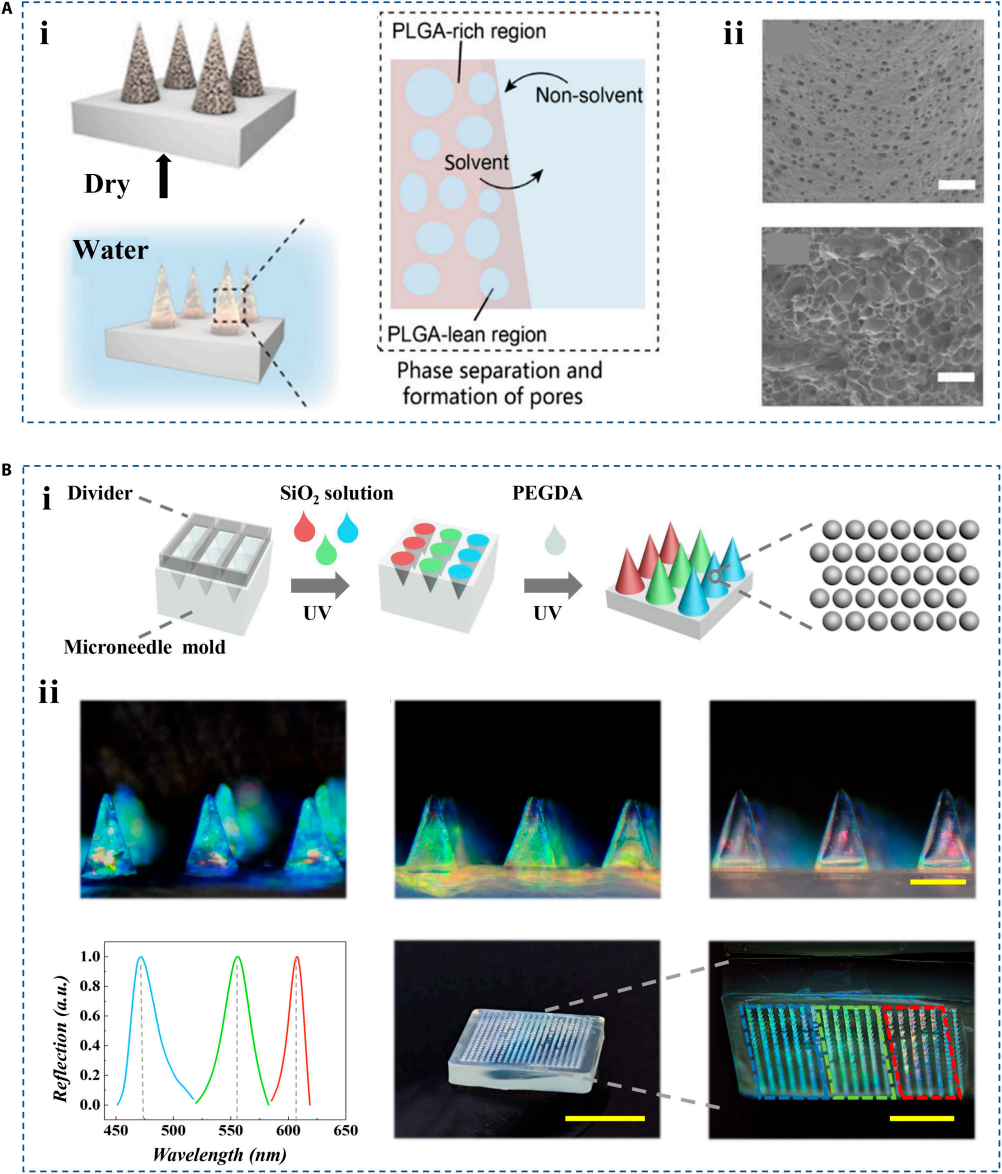
Fabrication of hierarchical biomaterials by micromanufacturing. (A) (i) The preparation schematic diagram and (ii) characterization of porous PLGA microneedles [70]. Copyright 2023, Wiley-VCH GmbH. (B) (i) The preparation schematic diagram and (ii) characterization of a non-closely packed colloidal crystal microneedle patch with optical and partition properties [144]. Copyright 2023, Wiley-VCH GmbH.

Micromanufacturing mainly refers to the fabrication of some micro- and nano-scale biomaterials with the help of molds, which requires researchers to design molds in advance. Among the many micromanufacturing materials from molds, microneedles are one of the most unique and outstanding ones in the field of wound healing. Microneedles have micron-sized spikes that can penetrate the barriers formed by wound clots, bacteria-derived biofilms, or scar tissue to deliver effective drugs or bioactives to deeper wound areas [66–69]. The researchers were able to fabricate porous hierarchical microneedles based on microneedle molds. For example, Ling et al. [70] used polylactic-coglycolic acid (PLGA) to develop a self-healing porous microneedle patch through low-temperature micro-molding and phase separation. In particular, PLGA was frozen at −20 °C to demold and stored in deionized water to form pores by phase separation. As the phase separation temperature increased from 4 to 37 °C, the diffusion rate of the solution and the rearrangement of the PLGA chains in the nanoparticles accelerated. The resultant microneedles had interconnected pores for drug loading (Fig. 7A). Wang et al. [71] used the sacrificial template method to prepare porous microneedles with inverse opal pores. The template here consisted of silica nanoparticles and was removed by hydrofluoric acid corrosion. The preparation of hierarchical structure based on the combination of microneedles and nanomaterials, such as liposomes, artificial vesicles, and metal-organic frameworks, is also a major focus to expand the functional microneedles in the field of wound healing [72,73]. Lu et al. introduced non-closely packed colloidal crystals formed by silica nanoparticles into microneedles. The final microneedle arrays had partitioned characteristics with encoded detection properties (Fig. 7B). Chen et al. developed microneedle bandages where dopamine-coated nanoparticles with selenium and chlorine E6 were introduced. The nanoparticles bidirectionally modulated the production of reactive species in response to the wound microenvironment [74].

## Curative Properties for Wound Healing

It has already been discussed before that repairing the wounds that are exposed to adverse conditions, such as the risk of bacterial infection and ongoing inflammation, is a difficult process. To address those difficulties, hierarchical biomaterials with highly curative properties, including drug delivery, antibacterial, antioxidant, angiogenesis, oxygenation, and wound monitoring, are proposed through the addition of special components or structural characteristics. In this section, an in-depth elucidation of how these properties contribute to the acceleration of wound healing will be provided. Additionally, exemplary illustrations of the distinctive strategies employed by hierarchical biomaterials to manifest these properties will also be presented.

### Drug delivery

**Fig. 8.**
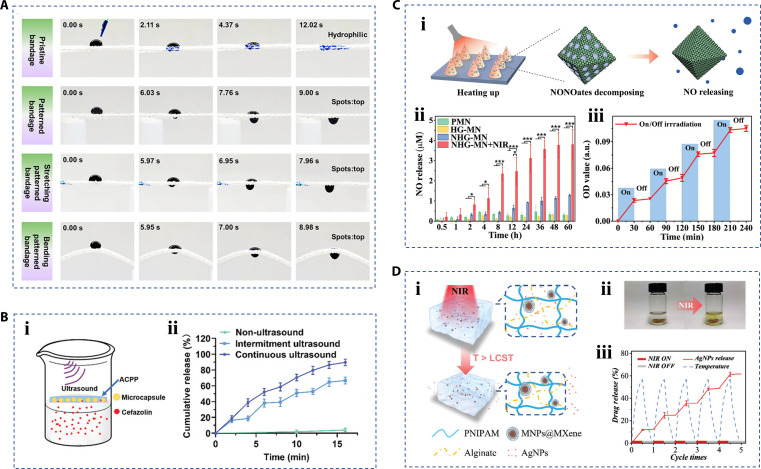
Hierarchical biomaterials with drug delivery property. (A) The characterization of the Janus hydrogel bandage with one-way drug delivery behavior [82]. Copyright 2024, American Chemical Society. (B) (i) The experimental schematic diagram and (ii) drug release statistics result of an ultrasonically responsive delivery system [86]. Copyright 2024, Wiley-VCH GmbH. (C) (i) The schematic diagram of microneedles for NO releasing, and (ii and iii) statistical results of NO release controlled by NIR light [90]. Copyright 2021, the authors. (D) (i) The NIR-controlled drug release schematic diagram and (ii) optical image of a hydrogel dressing based on (MNPs@MXene); (iii) AgNPs controlled release statistical result according to temperature changes [91]. Copyright 2021, Wiley-VCH GmbH.

Therapy administrations for chronic wounds usually utilize wound dressings to deliver drugs or bioactives to the wound site [75,76]. The porous structure in the hierarchical biomaterials provides ample space for the upload and delivery of drugs, and even controls the direction of drug diffusion [77–81]. For example, Wang et al. [82] presented the porous hydrogel bandage with Janus structure as a wound dressing. The researchers used gradient wettability channels to make the fluid move in one direction (Fig. 8A). This property gave the hydrogel bandage a unique continuous one-way drug delivery behavior from the outside to the wound area, effectively preventing drug leakage, contamination, or release in the incorrect direction. Unlike the preloading method, this one-way dosing nature also facilitated the substitution or supplementation of drugs to achieve a dynamic treatment regimen. In terms of drug delivery, controlled drug delivery has been proven to be beneficial to the phased treatment of wounds and reduce the side effects of drugs [83–85]. Different from the usual ways of controlling drug release through cross-linking agents or hydrolysis, hierarchical biomaterials can use internal response factors to release drugs under external stimulus. For example, Huang et al. [86] encapsulated antibiotics in poly(L-lactic acid) microcapsules, which were then integrated into poly(ethylene glycol) hydrogels as an ultrasonic responsive delivery system. Ultrasound has the advantages of noninvasiveness, tunability andstrong penetration in appilcation [87,88]. Microcapsules undergo expansion and contraction vibrations under ultrasound, so the release of drugs inside the microcapsule can be triggered and controlled by ultrasound. Experiments have shown that under continuous ultrasound, the hydrogel containing the microcapsule can release about 90% of the drug amount in a short time; in contrast, the release of antibiotics without ultrasound is less than 5%. Meanwhile, in an intermittent ultrasound treatment, the researchers also demonstrated that ultrasound accelerated the release of drugs, and once interrupted, the release of drugs immediately stopped (Fig. 8B). In summary, due to the focusing ability of ultrasound, the release of antibiotics could be controlled in time and space. In addition to ultrasound, near-infrared (NIR) light is also a common stimulus, which usually coordinates nanomaterials with photothermal response properties [89]. For example, Yao et al. [90] developed porous metal-organic scaffold microneedle patches containing graphene oxide. Benefitting from the temperature variation mediated by graphene oxide under NIR light irradiation, the nitric oxide (NO) contained in the microneedles achieved a controlled and responsive release (Fig. 8C). Similarly, Yang et al. [91] proposed a hydrogel dressing based on MXene-wrapped Fe_3_O_4_@SiO_2_ magnetic nanoparticles (MNPs@MXene) that could respond to both light and magnetism, which had advantages in terms of tissue penetration and high-sensitivity remote control. When exposed to NIR light, the temperature of the MNPs@MXene rose markedly, causing the hydrogel system to shrink, allowing for precise control of the drug release (Fig. 8D).

### Antibacterial

**Fig. 9.**
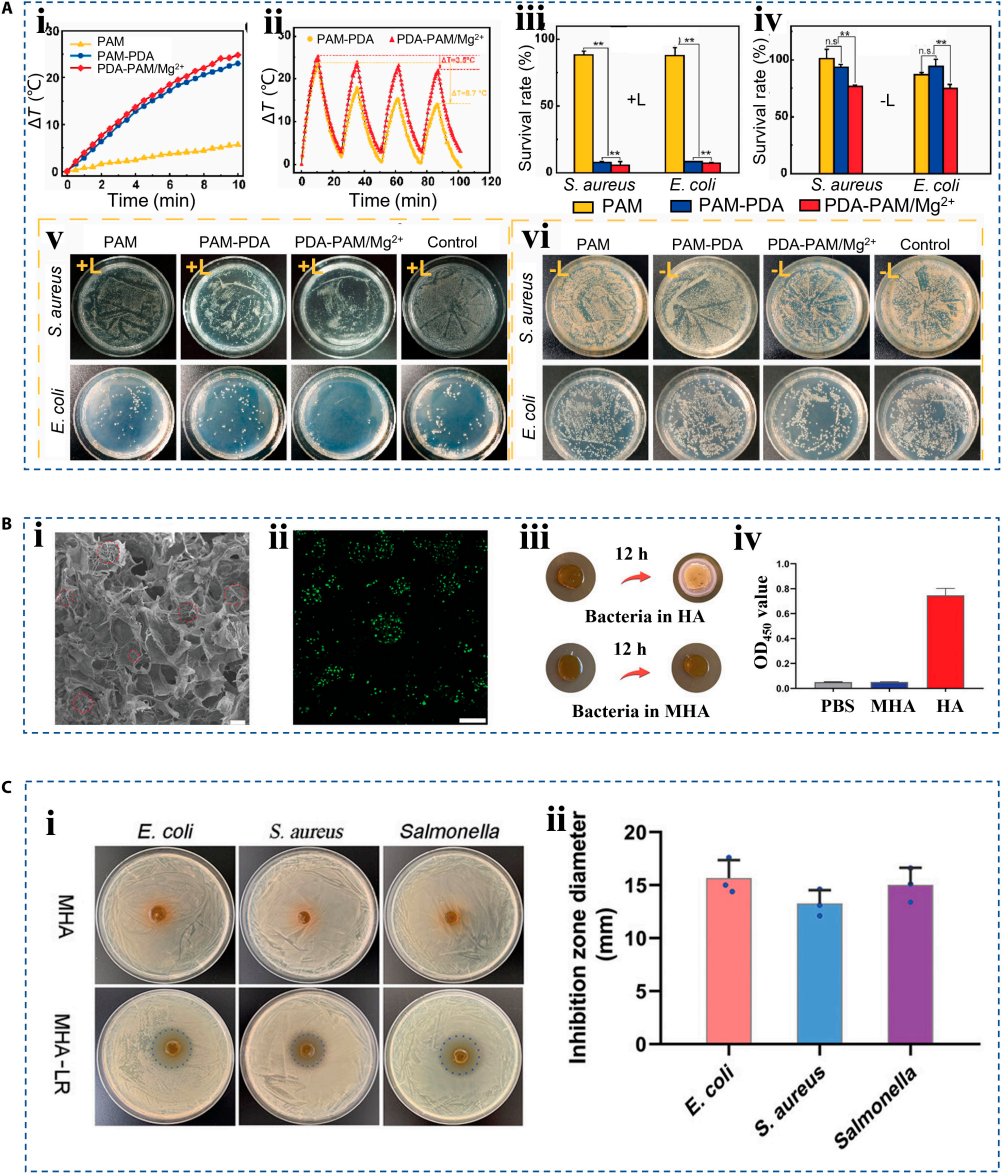
Hierarchical biomaterials with antibacterial property. (A) Statistical and image results of photothermal-induced antibacterial strategy: (i) Photothermal curves of different hydrogels. (ii) Temperature changes of the 2 hydrogels with repeated NIR. Survival rates (iii and iv) and images (v and iv) of *S. aureus* and *E. coli* after treatment with different hydrogels with (iii and v) and without (iv and vi) NIR [98]. Copyright 2021, the authors. (B) (i) The characterization of a hydrogel scaffold (MHA), (ii) the fluorescence image of live *L. reuteri* with MHA, (iii) the optical images showing MHA's prevention of bacterial escape, and (iv) the statistical result of escaping bacteria amount [99]. (C) (i) The optical images of antimicrobial result by employing *L. reuteri*, and (ii) the statistical result of inhibition zone diameter [99]. Copyright 2021, the authors.

Bacterial infection is a major obstruction to wound healing. The antibacterial property of hierarchical biomaterials was mainly imparted by 3 types of antimicrobial strategies. The first strategy is to add natural antimicrobial materials or nanozymes into the hierarchical biomaterials, such as copper, silver, zinc, and titanium dioxide [92–95]. In addition, some bioactives have also been reported. Gong et al. [34] paid attention to the insect cuticle proteins and developed a functional nanofibril film. The fibrous film presented an antibacterial effect similar to penicillin–streptomycin. The photothermal response of material is considered to be a powerful antimicrobial strategy [96]. Therefore, components with photothermal conversion effects are selected to be integrated into the hierarchical biomaterials including black phosphorus, polydopamine (PDA), and MXene [97,98]. Guo et al. [98] proposed a composite antimicrobial hydrogel with acrylamide, PDA, and magnesium ions as the core components. The addition of PDA imparted the hydrogel with a photothermal effect and photothermal stability, inducing a temperature increase of 14 °C (Fig. 9A, i). The subsequent addition of magnesium ions can improve the stability and repeatability of the photothermal effect, and the temperature variation can reach 21 °C (Fig. 9A, ii). PDA combined with magnesium ions reduced the survival rate of *Escherichia coli* and *S. aureus* to less than 10% in in vitro photothermal antibacterial test (Fig. 9A, iii to vi). As mentioned above, the microbiota in the wound plays a dual role. Traditional antimicrobial strategies may not only kill pathogenic bacteria but also inhibit the growth of beneficial bacteria, destroying the balance of the wound microbial system. Therefore, live bacterial therapy based on beneficial bacteria is considered a viable alternative treatment strategy. For instance, Ming et al. [99] reported a hydrogel scaffold containing *Lactobacillus reuteri*. *L. reuteri* is a beneficial bacterium that reduces the pH of the local environment by producing lactic acid and secreting the reuterin, an antimicrobial agent, to inhibit the growth of harmful bacteria. The authors encapsulated *L. reuteri* in microspheres via emulsion polymerization, which provided a scaffold for bacterial growth, protected the bacteria from the immune system, and prevented the bacteria from escaping into the surrounding environment (Fig. 9B). An in vitro antibacterial experiment showed that the prepared hydrogel produced obvious inhibition bands against *E. coli*, *S. aureus*, and *Salmonella* spp. on an agar plate, verifying its good antibacterial effect (Fig. 9C). This work may initiate a new approach to the management of wound infections using live bacteria, while its biological safety still requires further assessment.

### Antioxidant

**Fig. 10.**
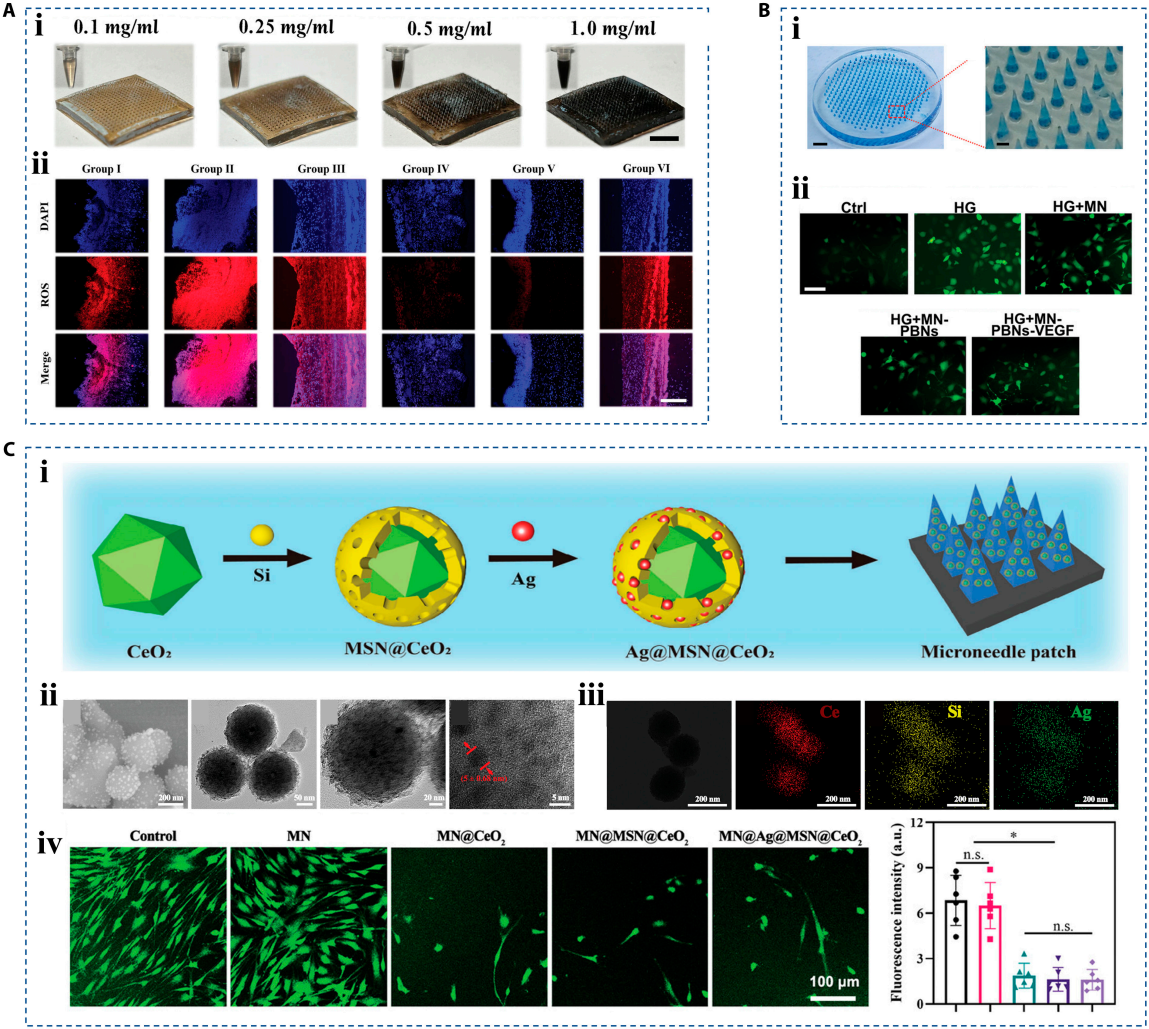
Hierarchical biomaterials with antioxidant property. (A) (i) Optical images of microneedle patches containing melanin nanoparticles with different concentrations, (ii) the antioxidant characterization of bacterial infected wounds in vivo, where Group I was the normal wound and Groups II to VI were the bacterial infected wound (Group II was the untreated bacterial infected wound, Group III received pure microneedle patch treatment, Group IV received antioxidant therapy by melanin-containing microneedle patch, Group V received antioxidant therapy by drug-loaded melanin-containing microneedle patch, and Group VI received drug therapy alone) [109]. Copyright 2024, Wiley-VCH GmbH. (B) (i) Optical images of microneedle patch containing Prussian blue nanozyme (MN-PBN) and (ii) the in vitro antioxidant characterization, where HG implies hyperglycemic treatment, and ROS was expressed as green fluorescence by DCFH-DA probe [110]. Copyright 2022, Wiley-VCH GmbH. (C) (i) Schematic diagram of a nanocomposite-loaded microneedle patch. (ii) SEM and TEM images and (iii) elemental mapping of synthesized cerium dioxide nanoparticles (CeO_2_), and (iv) the antioxidant characterization and statistics of in vitro cell experiments; ROS was expressed as green fluorescence by DCFH-DA probe [111]. Copyright 2024, Wiley-VCH GmbH.

In chronic wounds, the microenvironment with high ROS levels, inflammatory responses, and the resulting excessive oxidative stress can harm cell proliferation and differentiation, which leads to the deterioration of other functional cells at the wound site [100–102]. Therefore, the utilization of antioxidant materials to relieve oxidative stress and alleviate inflammatory response is a promising direction for promoting wound healing [103–105]. There are many natural antioxidant ingredients, such as tea polyphenols, tannic acid, platycodins, and melanin [100,106–108]. For example, Lu et al. [109] proposed a melanin-integrated inverse opal hydrogel microneedle patch with great antioxidant effect (Fig. 10A, i). Melanin nanoparticles extracted from cuttlefish were encapsulated in the 3D space of the hydrogel microneedles, which imparted antioxidant properties to the microneedle patch. The results of in vivo ROS staining showed that the intensity of red fluorescence representing ROS in the wounds of mice treated with melanin-containing microneedle patches was markedly reduced (Fig. 10A, ii). This result proved that the hierarchical materials containing melanin nanoparticles or other bioactives would have good antioxidant properties for wound treatment. In addition to natural ingredients, synthetic nanomaterials can also be integrated into the hierarchical materials to exert their antioxidant capabilities. Prussian blue nanozyme, a kind of Prussian blue complex in nanoform (<100 nm), is an antidote to phenoxy poisoning approved for clinical use, exerting its therapeutic effects by scavenging ROS and inhibiting inflammation. Guan et al. [110] developed a multifunctional hydrogel microneedle patch, which embedded Prussian blue nanozyme at the tip (Fig. 10B, i). To explore the antioxidant property of the microneedle patch, the researchers constructed a model of NIH-3T3 cells with high expression of ROS treated with high glucose and the oxidative stress inducer H_2_O_2_, respectively. The results of DCFH-DA (ROS probe) staining showed that the oxidative stress level of cells treated with a microneedle patch containing Prussian blue nanozyme was inhibited, indicating the antioxidant stress effect of this strategy (Fig. 10B, ii). Besides, Yu et al. [111] utilized cerium dioxide nanoparticles to fabricate a nanocomposite-loaded microneedle patch, which could scavenge excess ROS by mimicking the behavior of oxygen vacancies in superoxide dismutase and catalase through the redox cycle reaction between Ce^3+^ and Ce^4+^ (Fig. 10C).

### Angiogenesis

**Fig. 11.**
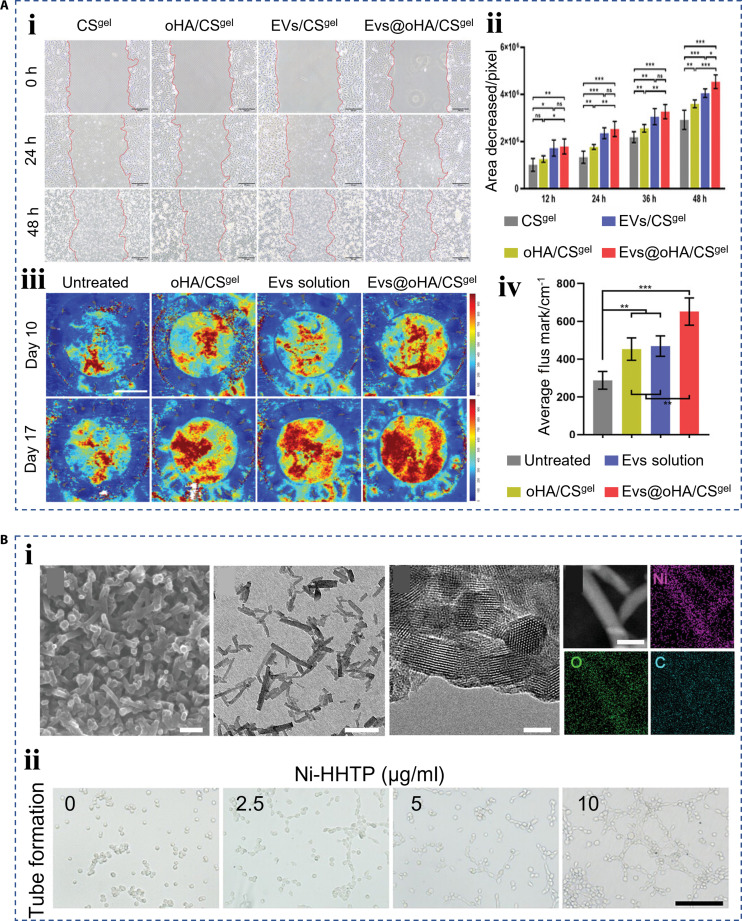
Hierarchical biomaterials with angiogenesis property. (A) (i) In vitro scratch experiment and (ii) the statistical result, (iii) in vivo blood flow images in wound sites, and (iv) the statistical result (CS^gel^ means chitosan hydrogel; oHA/CS^gel^ means chitosan modified with hyaluronic oligosaccharide; EVs means extracellular vesicles) [117]. Copyright 2023, Wiley-VCH GmbH. (B) (i) The characterization of Ni-HHTP, and (ii) the in vitro tube formation cell experiments with different concentrations of Ni-HHTP [121]. Copyright 2023, Wiley-VCH GmbH.

Due to the long-lasting inflammatory response in chronic wound, the angiogenesis of wound tissue is impaired, which may decrease the nutrient exchange and oxygen supply in the wound site, forming a vicious circle [30]. Therefore, promoting the angiogenesis of the hierarchical biomaterial is also a vital property for wound tissue [112,113]. As angiogenesis is mainly regulated by cytokines or cell-derived actives, such as vascular endothelial growth factor [114], recombinant human epidermal growth factor [115], and exosomes [116], cytokine supplementation toward wounds is the mainstream therapeutic strategy. For instance, a hemangioma stem cell-derived extracellular vesicle with angiogenic activity was proposed as a bioactive by Lu et al. [117]. Meanwhile, they fabricated a porous hydrogel carrier of hyaluronic oligosaccharide-modified chitosan to encapsulate the extracellular vesicles. Hyaluronic oligosaccharide is a degradation product of hyaluronic acid, which can activate steroid receptor coactivators, focal adhesion kinases, and extracellular signal-regulated kinases. It has been proven to have a certain role in promoting angiogenesis as well. Researchers utilized transwell coculture to explore how the biomaterial behaved in scratch experiments in vitro. The results showed that the extracellular vesicles released from the hydrogel could significantly improve the proliferation and migration of human umbilical vein endothelial cells and had a significant angiogenetic effect (Fig. 11A, i and ii). In the animal test, laser Doppler flowmeters were used to measure blood flow at the wound site, and the results demonstrated the combined roles of extracellular vesicles and hyaluronic oligosaccharide in promoting angiogenesis (Fig. 11A, iii and iv). In addition to cytokines, some artificial nanozymes are also of interest to promote angiogenesis [118,119]. Drawing inspiration from the intricate architecture and catalytic prowess of natural enzymes, metal-organic frameworks, which are crystalline coordination networks meticulously assembled from metal ions/clusters and organic ligands, have emerged as a promising class of artificial enzyme mimics [120]. The well-defined periodic arrangement of metal nodes and organic linkers within metal-organic frameworks endows these materials with highly ordered porous structures and multichannel frameworks. This unique structural characteristic enables the catalytically active sites of nanozymes to be optimally exposed, facilitating efficient mass transfer and intimate interaction with substrates. As a result, metal-organic framework-based nanozymes can effectively recapitulate the catalytic mechanisms of natural enzymes, offering potential for a wide range of catalytic applications. For example, Liu et al. [121] fabricated a metal-organic framework based on Ni (called Ni-HHTP) (Fig. 11B, i). Ni ion has been shown to enhance angiogenesis through up-regulating the expression of vascular endothelial growth factor and activating the transforming growth factor-β signaling pathway. In vitro cell experiments have also validated its excellent performance in facilitating cell proliferation, migration, and tube-like structure formation (Fig. 11B, ii). Besides, the angiogenesis promotion of Ni-HHTP was also verified in the in vivo animal experiment. These results demonstrated that the combination of those kinds of artificial nanozymes with hierarchical biomaterials was highly promising for the treatment of refractory wounds.

### Oxygenation

**Fig. 12.**
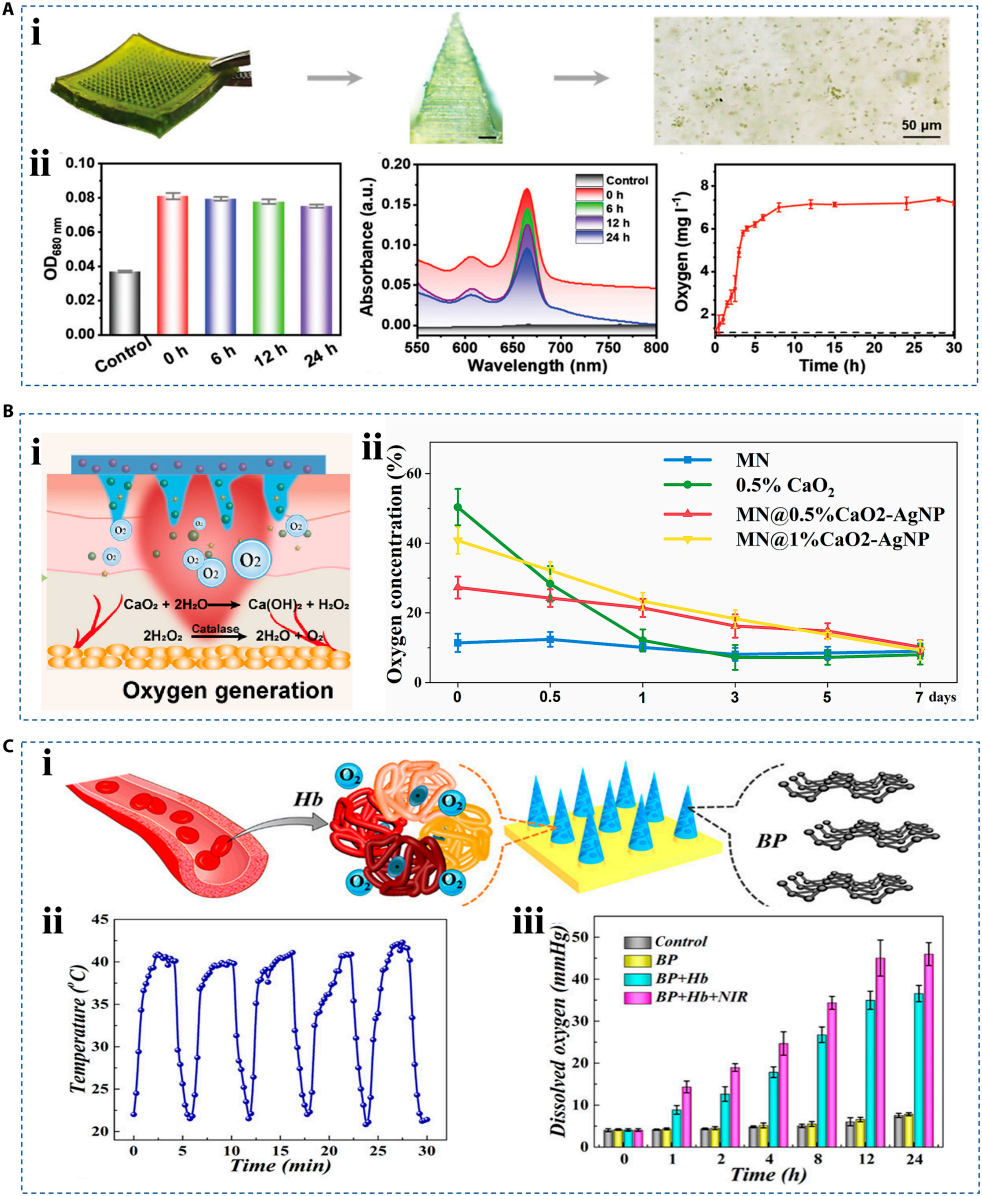
Hierarchical biomaterials with oxygen supply property. (A) (i) Optical image of the microneedle patch loaded with chlorella, and (ii) the activity of chlorella within 24 h and its statistical curve of oxygen release within 30 h [126]. Copyright 2024, Wiley-VCH GmbH. (B) (i) Oxygen production schematic diagram of the microneedle patch with a needle tip containing both calcium oxide and catalase, and oxygen release curve in an anoxic environment, and (ii) statistical results after oxygenation by microneedle patch [128]. Copyright 2024, the authors. (C) (i) Schematic diagram of oxygen supply by loading hemoglobin and black phosphorus, (ii) heating behavior of microneedles under NIR, and (iii) oxygen release statistics controlled by NIR irradiation [129]. Copyright 2020, American Chemical Society.

Hypoxia is an important pathological feature of the chronic wound microenvironment [122]. The traditional therapeutics of oxygen supply still face the challenge of poor stability and biosafety, as well as the inefficient oxygen transport and utilization [123]. In contrast, in situ oxygen production is proposed for a novel oxygenation strategy. In recent studies, algae were considered biocompatible oxygen-making donors that can produce dissolved oxygen in situ upon photosynthesis [124,125]. For example, Gao et al. [126] proposed a microneedle patch loaded with chlorella to enable efficient oxygen transport. Chlorella is a natural oxygen-producing organism that allows for sustainable and controlled oxygen production. The researchers demonstrated that chlorella remained active in the microneedle patch for at least 24 h by detecting chlorophyll content. Meanwhile, the microneedle patch could produce oxygen for up to 30 h under light conditions (Fig. 12A). In addition to oxygen-producing microorganisms, it is also possible to integrate oxygen-producing chemical reaction feedstocks into hierarchical materials [127]. Sun et al. [128] fabricated a microneedle patch with a needle tip containing both calcium oxide and catalase. Calcium oxide was used to react with water in the wound to generate calcium hydroxide and hydrogen peroxide, and the presence of catalase can decompose hydrogen peroxide into the required oxygen to effectively inhibit the production of ROS in the oxygen production process (Fig. 12B). Zhang et al. [129] proposed another oxygen delivery system as an optimization of oxygen transport. They integrated black phosphorus with the oxygen-carrying protein hemoglobin into the tip of the microneedle patch. The microneedle patch was first immersed in an oxygen-enriched solution to take in sufficient oxygen, which depends on the amount of encapsulated oxygen-carrying protein hemoglobin. Then, based on a photothermal conversion device combining NIR and black phosphorus, the oxygen release could be controlled. The increase in temperature would weaken the oxygen-binding capacity of the hemoglobin, promoting the release of oxygen (Fig. 12C).

### Wound monitor and sensor

**Fig. 13.**
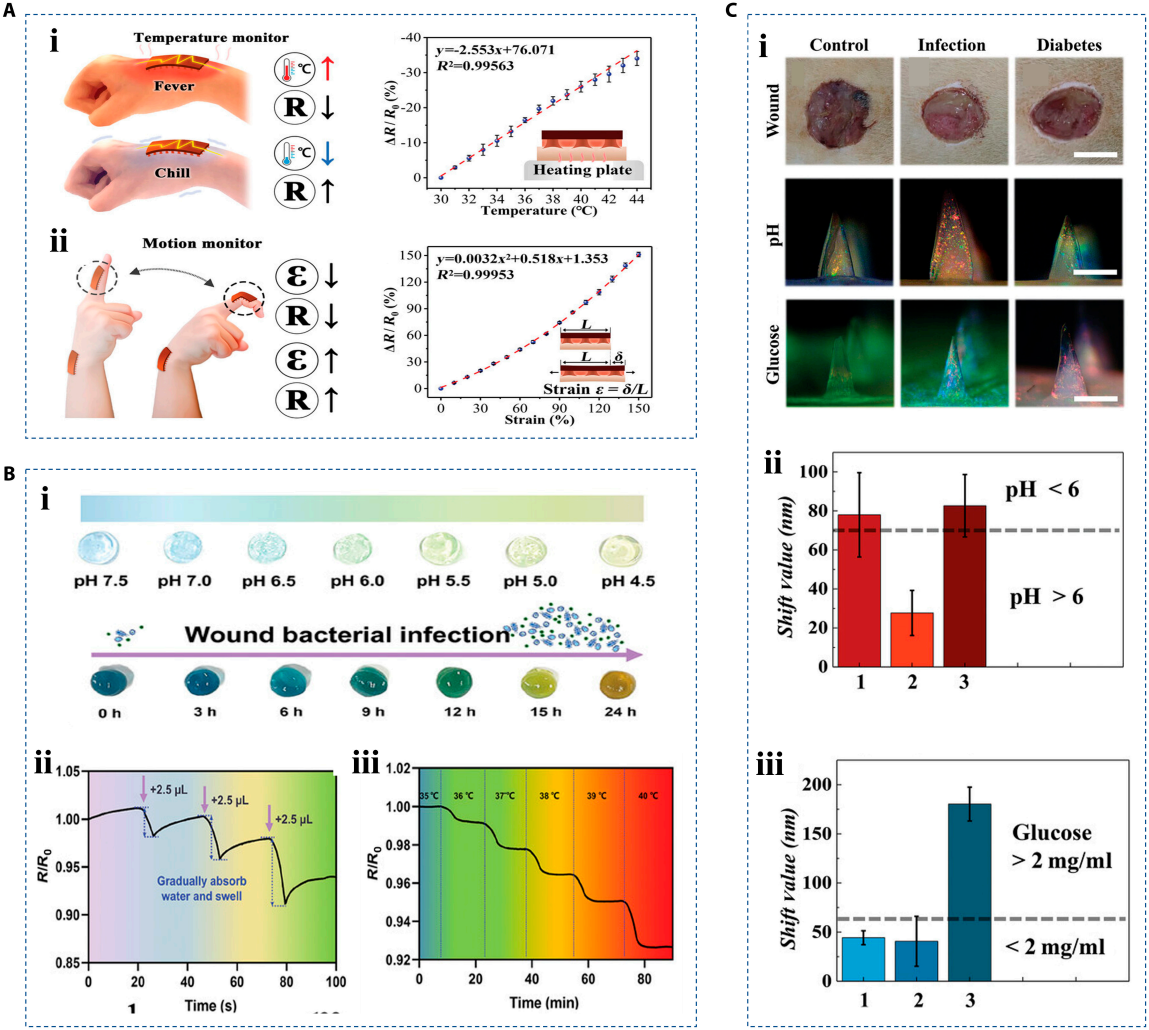
Hierarchical biomaterials with a monitor and sensor property. (A) Ag-tannin nanoparticle-loaded patches with (i) temperature and (ii) tensile dual-response [138]. Copyright 2023, the authors. (B) Hydrogels with (i) pH colorimetric detection of bacterial infections, (ii) electrical detection of exudate, and (iii) temperature detection [139]. Copyright 2024, the authors. (C) (i) In vivo detection images of coded microneedles, and (ii and iii) statistical results [140]. Copyright 2023, Wiley-VCH GmbH.

With meticulous design, hierarchical materials can not only promote wound healing but also realize wound monitoring or sensing after combining with detectable circuits. This function helps to reflect the wound condition in real time, providing a basis for clinical diagnosis and subsequent treatment [130,131]. Wound temperature, pH, uric acid, glucose concentration, and exudate are the key objects of wound detection [132–134]. For example, temperature measurement can assist in estimating wound status [135]. Change of pH can reflect wound bacterial infection [136]. Glucose concentration has guiding significance for diabetic ulcers [137]. Benefiting from the complexity of the structure and contents, hierarchical biomaterials have emerged to achieve multiple detection functions instead of single-index detection. Nanomaterials with good conductivity can be introduced into the hierarchical materials for various detections by a change in resistance. For example, Ag-tannin nanoparticle was used to feed back the temperature and strain (Fig. 13A) [138]. Similarly, Lei et al. [139] designed a nanocomposite hydrogel with simultaneous pH detection and temperature sensing as well as exudate detection capabilities. Bromophenol blue was added to the hydrogel as a pH indicator. The color of the hydrogel would change when incubated with different densities of *S. aureus* cultures in the microenvironment at different growth stages, which demonstrated that the hydrogel had the potential to feed back bacterial infection (Fig. 13B, i). Then, the electrical conductivity of the hydrogel itself was used to reflect the temperature change of the wound environment and the increase of exudate (Fig. 13B, ii and iii). Similarly, Lu et al. [140] prepared a coded microneedle patch with simultaneous pH, glucose, and histamine detection capabilities based on the characteristics of non-closely packed colloidal crystals and hydrogels. The hydrogel volume would shrink or swell when the combination of hydrogel and target molecules occurred, and the spacing of nanoparticles in the colloidal crystal would change, which finally caused an obvious structural color change and shift of the reflection spectrum of the colloidal crystal. This strategy, by color change visible to the naked eye, had an advantage over the resistance-based detection method that requires external connection measuring, which had the potential to realize in situ and convenient wound detection (Fig. 13C).

It is remarkable that although we classify the properties of hierarchical biomaterials separately, most of them are not limited to a specific type of required properties. For the complex microenvironment in wounds, a variety of combinations of the above properties are the practical strategies for hierarchical materials. For example, the combination of antibacterial, antioxidant, and angiogenesis can accelerate wound healing commendably. Meanwhile, these characteristics are interlinked and mutually reinforcing. For instance, oxygenation to the wound could further have an antioxidant effect, finally effectively promoting angiogenesis in the tissue. This requires the further integration of multiple types of components in the hierarchical biomaterial.

## Applications

In addition to the properties required to address the difficulty of wound healing, hierarchical biomaterials put forward new characteristic requirements when applied to wound sites of different tissues or organs. In this section, we will focus on the aspects of skin wound healing, oral wound healing, gastric wound healing, and ocular wound healing. The unique functions given to hierarchical biomaterials in these special application environments and how to achieve them are also discussed (Table 1).

**Table 1.** Application of hierarchical biomaterials in different wounds

Wound type	Tissue structure	Wound characteristics	Advantages of hierarchical biomaterials	Reference
Skin wound	1. Epidermis: containing the keratinocytes. 2. Dermis: composed of collagen fibers, fibroblasts, blood vessels, and nerve endings. 3. Subcutaneous tissue: primarily consists of fat, blood vessels, and nerves.	Excessive scar hyperplasia	1. Promote the cell activity of fibroblasts and keratinocytes. 2. Control wound inflammation and hypoxia, and alleviate the formation of scars. 3. Promote active wound closure.	[146,149,155]
Oral wound	1. Epithelial layer: composed of stratified squamous epithelium. 2. Lamina propria: rich in blood vessels, nerves, and salivary glands, and also contains immune cells such as lymphocytes.	Moist environment; frequent mechanical stimuli such as daily chewing and speaking	1. Strong adhesion and long duration. 2. Antibacterial, antioxidant, angiogenesis-promoting, and scavenge bacterial biofilm by adding hierarchical materials.	[161–166]
Gastric wound (e.g., ulcer)	1. Mucosal layer: composed of simple columnar epithelium, containing mucous cells that secrete protective mucus. 2. Submucosa: distributes blood vessels and nerve plexuses. 3. Muscularis propria: composed of smooth muscle, whose main function is to realize gastric peristalsis.	Gastric acid and pepsin; in the body	1. High adhesive ability and stomach acid resistance. 2. Injectable properties and in situ formation. 3. Good antibacterial and hemostatic performance.	[167,170,174]
Ocular wound (e.g., corneal injury)	1. Cornea: a 5-layer structure with no blood vessels distributed in the cornea. 2. Conjunctiva: rich in blood vessels, containing goblet cells that secrete mucus.	Affect vision; low bioavailability	1. Mechanical stability and transparency 2. Inhibit corneal interstitial fibrosis. 3. Easy adhesion and high bioavailability by microneedle.	[181,182,184,185]

### Skin wound healing

**Fig. 14.**
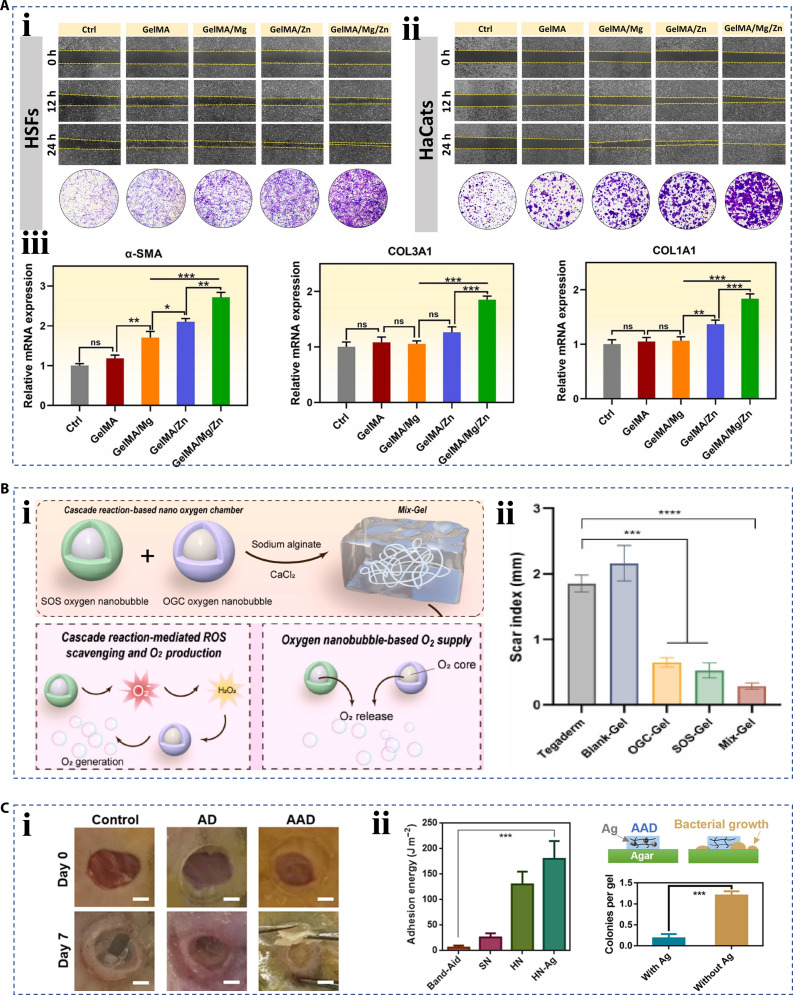
Hierarchical biomaterials for skin wound healing. (A) The in vitro migration test of (i) human skin fibroblasts (HSFs) and (ii) human keratinocytes (HaCats), and the expression results of (iii) α-SMA, COL3A1, and COL1A1 [142]. Copyright 2023, the authors. (B) (i) Schematic diagram of nanocarrier-loaded hydrogel dressing based on a cascade enzyme reaction, and (ii) the statistical result of wound scar index [145]. Copyright 2024, the authors. (C) (i) The optical images of wounds treated by AAD for contraction. (ii) The characterization of adhesion and antibiosis by adding Ag nanoparticles [151]. Copyright 2019, the authors.

Skin represents the most voluminous organ within the human anatomical structure, and it is often injured by external mechanical force. The main cellular components of human skin are fibroblasts and keratinocytes, whose proliferation and migration in the wound site are beneficial to promote wound healing [14,141]. For example, Yang et al. [142] proposed a GelMA hydrogel dressing loaded with zinc and magnesium particles, as zinc and magnesium ions have been proven to be involved in cellular activity. In vitro experiments have shown that the synergistic effect of the 2 ions could significantly promote the migration of fibroblasts and keratinocytes, as well as the transformation of fibroblasts into myofibroblasts (Fig. 14A). Hence, the production and remodeling of the extracellular matrix were accelerated. In addition, with the increasing emphasis on beauty, the absence of scarring during the healing process of skin wounds is also one of the functions required for dressing materials. Hypoxia and excessive ROS are major barriers to wound healing after trauma, which resulted in keloids and hypertrophic scars [143,144]. Therefore, Han et al. [145] developed a nanocarrier-loaded hydrogel dressing (Mix-Gel) based on a cascade enzyme reaction (Fig. 14B, i). The used nanocarriers included oxygen nanobubbles modified respectively with superoxide dismutase and catalase. The cascade reaction of the 2 nanocarriers promoted the superoxide conversion in the wound surface and finally converted it into oxygen, which effectively controlled wound inflammation and hypoxia, and alleviated the formation of scars (Fig. 14B, ii). Besides, transforming growth factor-β inhibitor, verteporfin, and youthful brain-derived extracellular vesicles have also been demonstrated to be effective in inhibiting scar formation in the latest research [146–149]. Human skin healing mainly depends on re-epithelialization, and there is a lack of active contraction, which prolongs the wound closure time [150]. Therefore, inspired by the ability of embryonic wound contraction, Blacklow et al. [151] proposed a new active adhesion dressing (AAD) that could exert sufficient contractile force to promote active wound closure (Fig. 14C, i). The dressing consists of poly(*N*-isopropyl acrylamide) (PNIPAm), Ag nanoparticles, and alginate. The thermal response property of PNIPAm endowed the dressing ability of temperature-triggered contraction. The addition of Ag nanoparticles not only provided antimicrobial properties but also improved the adhesion of the dressing (Fig. 14C, ii).

### Oral wound healing

**Fig. 15.**
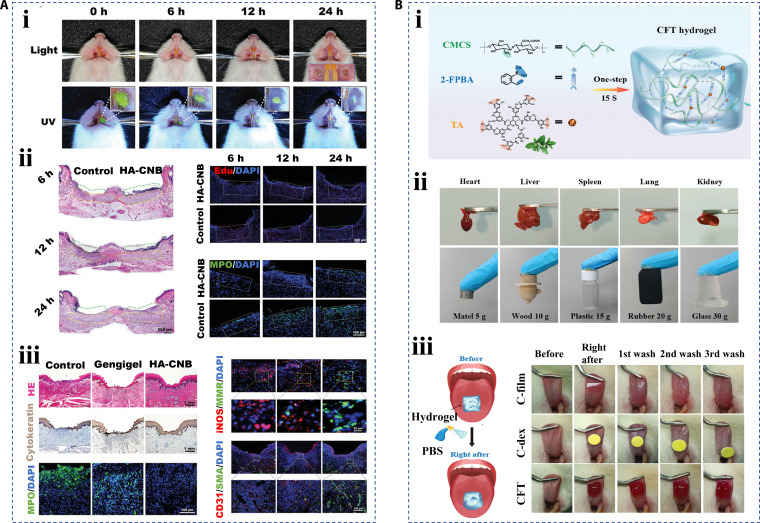
Hierarchical biomaterials for oral wound healing. (A) (i) The observation of HA-CNB hydrogel remaining in the rat oral cavity, (ii) the characterization of HA-CNB hydrogel for promoting oral wound healing in rats, and (iii) in porcine [157]. Copyright 2021, Wiley-VCH GmbH. (B) (i) Formation schematic diagram of CFT hydrogel based on a dual-dynamic covalent cross-linking strategy, (ii) the characterization of CFT hydrogel adhesion property on different tissues and substrates, and (iii) the adhesion property comparison of CFT hydrogel to other commercial control agents (C-film, C-dex) on rat tongue [158]. Copyright 2024, Wiley-VCH GmbH.

The oral cavity is another common place where wounds occur in daily life, caused by mouth ulcers and dental surgery, among others [152,153]. The oral wound healing process takes place in a unique warm, humid environment containing millions of microorganisms [154,155]. Therefore, the most important requirement for hierarchical materials in this particular application is that the material must have excellent wet tissue adhesion property to overcome obstacles caused by humid environments, saliva flushing, and oral movements [156]. Considering that, Zhang et al. [157] proposed a photo-cross-linked adhesive hydrogel (HA-CNB). Unlike other agents commonly used in clinical practice with weak adhesion and short duration, this elastic and degradable hyaluronic acid gel could achieve mucosal protection for more than 24 h (Fig. 15A, i). The results of rat and porcine oral mucosal repair models showed that this novel adhesive gel created a favorable microenvironment where tissue repaired faster and better (Fig. 15A, ii and iii). Su et al. [158] proposed the injectable porous hydrogel (CFT hydrogel) with adhesion ability from a dual-dynamic covalent cross-linking strategy, which had the advantages of rapid prototyping, simplicity, and high bioavailability. The first layer of dynamic covalent cross-linking was built by 2-formylphenylboronic acid and carboxymethyl chitosan in hydrogels based on their respective amino and aldehyde groups. After that, with the addition of tannin, the boric acid group on the 2-formylphenylboronic acid and the phenol group on the tannin successfully constructed the second dynamic cross-linking (boric acid bond) (Fig. 15B, i). The strong adhesion of hydrogel is largely attributed to the dense phenolic groups within tannin. These phenolic groups allow the hydrogel to stick firmly to different substrates through noncovalent interactions, such as hydrogen bond superposition and electrostatic interaction. The researchers demonstrated the potential of the hydrogel’s excellent wet adhesion property and practical applications through adhesion to different tissues and in vitro experiments (Fig. 15B, ii and iii). In addition to adhesion, the introduction of suitable therapeutic components into the hydrogel system is another advantage that can be achieved with hierarchical biomaterials. For example, tea polyphenol is a natural ingredient with good antibacterial and antioxidant properties, which have been demonstrated in oral wound treatments [159]. Bone marrow mesenchymal stem cells could be added into a hydrogel system, and the biocomponents, such as growth factors released by them, could exert anti-inflammatory and angiogenesis-promoting effects [160,161]. Liu et al. [162] presented copper-doped carbon dots to scavenge bacterial biofilm.

### Gastric wound healing

**Fig. 16.**
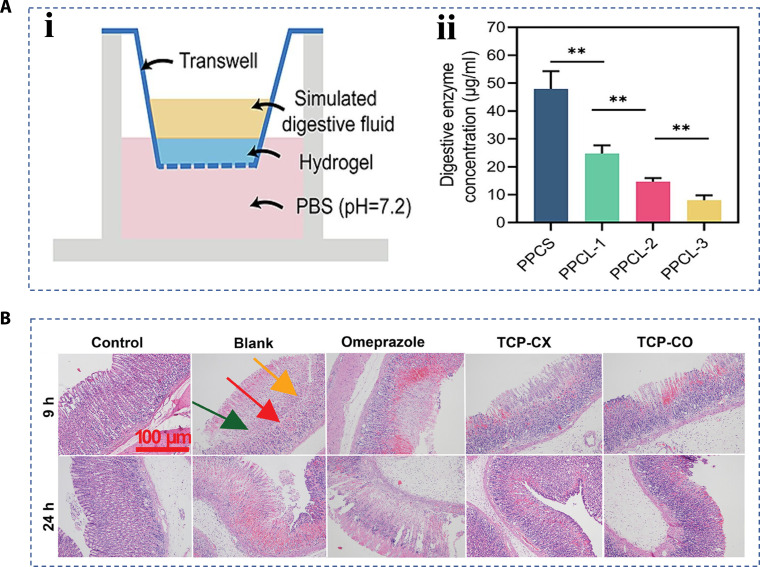
Hierarchical biomaterials for gastric wound healing. (A) (i) Schematic diagram and (ii) result of anti-gastric acid test [166]. Copyright 2024, Elsevier B.V. (B) The H&E characterization of gastric ulcer healing in rats, in which the green arrow pointed to gastric mucosal epithelial cells, the red arrow pointed to bleeding, and the yellow arrow pointed to neutrophilic inflammatory cell infiltration [163]. Copyright 2024, Wiley-VCH GmbH.

Represented by a gastric ulcer, a gastric wound is also a common type of wound [163]. In addition to the same moist environment and tissue peristalsis as in the mouth, the gastric acid specifically secreted by the stomach and the resulting acidic environment pose a new challenge to hierarchical materials [164,165]. Ni et al. [166] proposed a new adhesive hydrogel for repairing gastric ulcers, which had key functions including high adhesive ability and stomach acid resistance. Tissue adhesion of hydrogel consisting of polyethyleneimine, polyacrylic acid, and modified chitosan was achieved by hydrogen bonding and ion interaction, and the strong intermolecular force made the hydrogel exhibit excellent impact resistance. Besides, the researchers designed a transwell experimental model to explore how the hydrogel behaved in an isolated stomach acid environment. The result revealed that the downward penetration of digestive enzymes in the upper cavity was reduced, which proved that the hydrogel could isolate the digestive environment from the stomach and help protect the smooth healing of the ulcer site (Fig. 16A). Unlike the skin and mouth, gastric wounds are difficult to treat with conventional ways because they occur inside the body. Therefore, there is a requirement for hierarchical materials with injectable properties and in situ formation. Specifically, injectable hydrogels are pre-gel liquids with fluidity at room temperature that can undergo a transformation to form hydrogel dressings in a short period of time when they reach the wound site [167–169]. In one study, He et al. developed a sequence of hydrogels, which could self-heal after being injected and have adhesion capacities. These features were based on the free-radical polymerization between 6-aminocaproic acid monomer and its derivatives [170]. The excellent ability of hydrogel to stop bleeding and promote wound healing has been demonstrated by in vivo experiments on the porcine stomach. Coincidentally, the Wen et al. [163] prepared an injectable hydrogel based on carboxymethyl chitosan and introduced thrombin-derived C-terminal peptide (TCP-25). This TCP-25 peptide hydrogel could adapt to the gastrointestinal microenvironment and had the required characteristics of acid resistance and high adhesion, among others. Benefitting from the material properties, the TCP-25 peptide hydrogel had showed good antibacterial and hemostatic performance, and promoted wound healing (Fig. 16B).

### Ocular wound healing

**Fig. 17.**
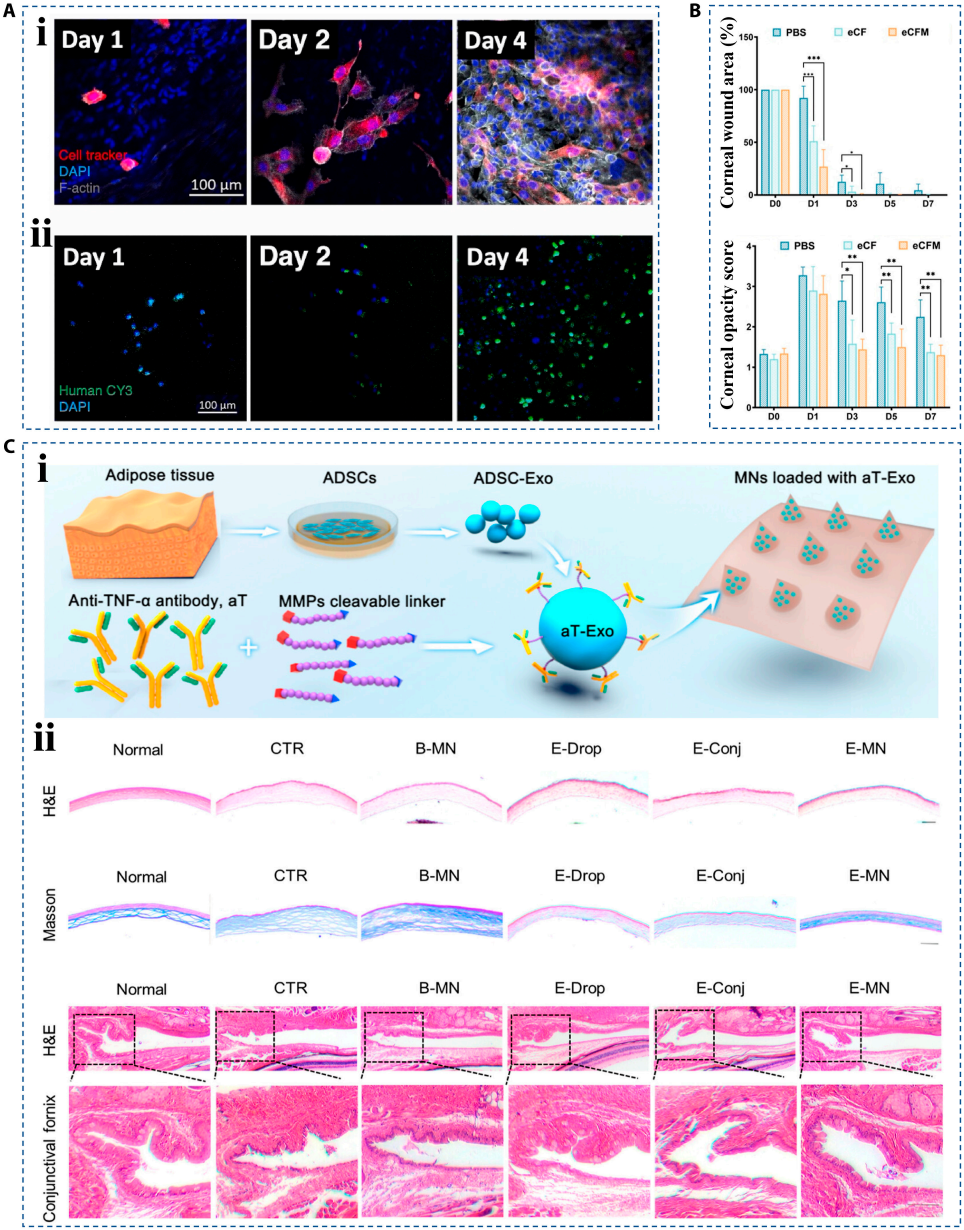
Hierarchical biomaterials for ocular wound healing. (A) The characterization of hydrogels loaded with corneal cell for promoting (i) adhesion and (ii) proliferation in rabbit eye [177]. Copyright 2021, Elsevier Inc. (B) The statistical results of collagen fibers for promoting ocular wound healing in rats and reducing fibrosis, where eCF means electrospun collagen nanofibers and eCFM means eCF membrane [178]. Copyright 2024, American Chemical Society. (C) (i) Schematic diagram of microneedles encapsulating antibody-modified exosomes, and (ii) the characterization of microneedles to treat ocular wounds, where CTR means no treatment, B-MN means blank microneedles, E-drop means aT–Exo eyedrop, E-Conj means aT–Exo subconjunctival injection, and E-MN means aT–Exo loaded microneedles [181]. Copyright 2024, American Chemical Society.

Corneal epithelium is the barrier of the eye that resists external environmental fluctuations and environmental damage, keeping the smooth surface of the eye [171]. As a result, corneal trauma or concurrent infections can lead to severe eye pathology and even blindness [172,173]. Traditional eye drops, although they are the mainstay of treatment for corneal wounds, have low bioavailability [174,175]. To address this challenge, Zhao et al. [176] developed a composite hydrogel loaded with rutin to form contact lenses, which could release rutin for a long time and promote corneal wound healing. Fernandes-Cunha et al. [177] designed a hydrogel hierarchical material encapsulated with corneal cells, which was based on hyaluronic acid. The hydrogel had mechanical stability and transparency adapted to the cornea. In addition, as a cell delivery vehicle, porous hydrogels had good biocompatibility and could also spread cells, transporting cells to the eye and promoting cell adhesion and proliferation on the cornea (Fig. 17A). Zhang et al. [178] studied the potential of collagen in ocular wound healing. They fabricated electrospinning collagen nanofibers, which maintained the natural characteristics of collagen and had high porosity. It demonstrated that the collagen nanofibers could facilitate cell proliferation and migration, and inhibit the production of inflammatory cytokines. Therefore, this material would play a therapeutic role in inhibiting corneal interstitial fibrosis and promoting ocular wound healing (Fig. 17B). In addition to hydrogel membranes, microneedles have been selected as drug carriers for corneal therapy due to their excellent transdermal delivery properties [179]. For example, Kong et al. [180] proposed a novel adhesive proanthocyanidin microneedle patch that could easily adhere to the injured area in a moist eye environment, and the antioxidant properties of the loaded proanthocyanidin could effectively scavenge ROS, which had been proven to promote healing in a rat model of ophthalmic burns. Further, Yu et al. [181] introduced adipose-derived mesenchymal stem cell exosomes into dissolving microneedles to treat ocular wounds (Fig. 17C, i). The researchers first modified exosomes with antibodies (aT–Exo) to improve their anti-inflammatory properties. At the same time, microneedles had the mechanical properties of being able to penetrate the cornea, which overcame the low osmosis in exosome drip therapy and improved the efficiency of exosome treatment (Fig. 17C, ii).

### Other applications

**Fig. 18.**
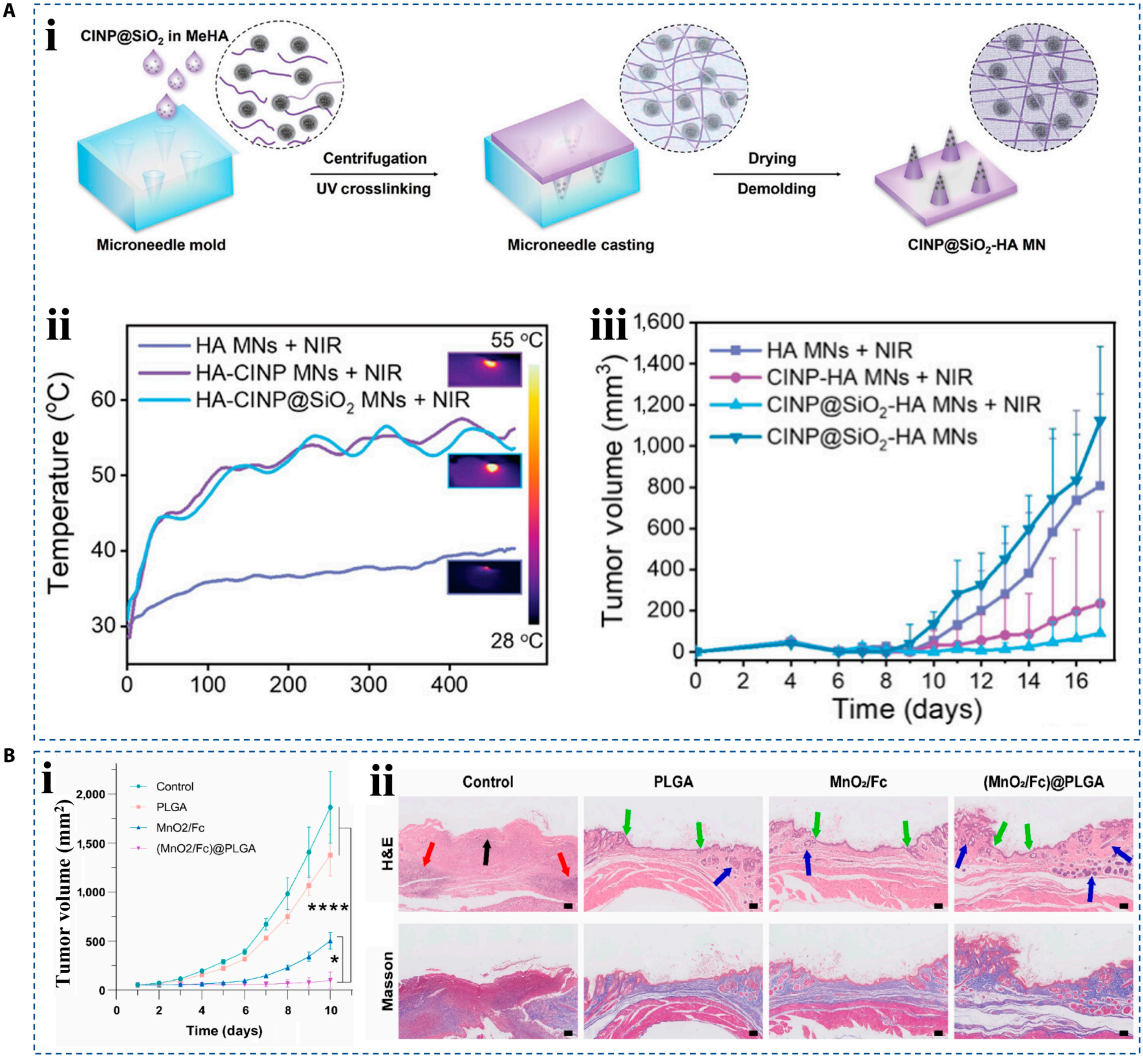
Hierarchical biomaterials for tumor surgery wound healing. (A) (i) Diagrammatic drawing of melanin-containing microneedle patch fabricating, (ii) photothermal response of microneedle patch, and (iii) tumor volume after photothermal antitumor therapy [185]. Copyright 2022, Wiley-VCH GmbH. (B) (i) Anti-tumor effects of chemodynamic therapy and (ii) characterization of wound healing. Black arrow: scab, red arrow: inflammatory infiltration, green arrow: epithelial tissue, and blue arrow: glands and hair follicles [186]. Copyright 2024, the authors.

In addition to wounds on different organs, wounds created after tumor resection surgery also require further treatment. For such wounds, the dressings need to promote wound healing and also need to have anti-tumor ability to prevent tumor recurrence after surgery [182–184]. Lei et al. [185] fabricated a hyaluronic acid hydrogel microneedle patch containing biomineralized melanin nanoparticles. The addition of melanin nanoparticles extracted from ink sacs had natural antioxidant and photothermal conversion capabilities, and their presence gave the microneedle patch the ability to scavenge ROS and realize tumor photothermal therapy. To validate the anti-tumor properties of patch, the researchers established a mouse model of melanoma resection. Tumor recurrence was observed on 9 days after surgery, and compared with the control group, most of the tumors in mice treated with microneedle photothermal treatment shrank or disappeared without recurrence and no damage to the surrounding tissues. This result demonstrated the potential of hierarchical materials with photothermal conversion properties in tumor postoperative wound healing (Fig. 18A). At the same time, Wang et al. [186] proposed a chemodynamic therapy-based anti-tumor hierarchical material strategy. Chemodynamic therapy refers to the inhibition of tumor growth by converting endogenous H_2_O_2_ into hydroxyl radicals through a Fenton or Fenton-like reaction under the action of a catalyst. The MnO_2_ nanoparticles and ferrocene Fc in the hydrogel can achieve efficient chemodynamic antitumor therapy by releasing Fe^2+^ and Mn^2+^, and had been verified in the postoperative wound treatment of murine osteosarcoma (Fig. 18B).

**Fig. 19.**
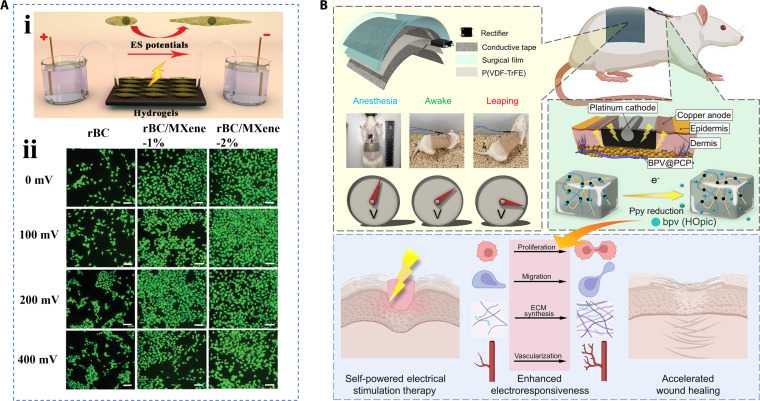
Hierarchical biomaterials with electrical stimulation for wound healing. (A) (i) Experiment diagrammatic drawing of electrical stimulation on NIH-3T3 cells, and (ii) the characterization of the number of cells and their viability under electrical stimulation culture [192]. Copyright 2020, Wiley-VCH GmbH. (B) Schematic diagram of an electrically responsive hydrogel system combined with a wearable DC pulsed piezoelectric nanogenerator for promoting wound healing [193]. Copyright 2023, American Chemical Society.

Even if they contain effective bioactives that promote wound healing, most wound healing dressings are mainly passively involved in the healing process. It has been shown that electrical stimulation can participate in the active healing of wounds by stimulating the behavior of skin cells [187,188]. When the epithelium is damaged, the wound site naturally generates an endogenous direct current electric field, which is thought to actively regulate cell proliferation, migration, and other cellular behaviors, and is the primary guiding signal to promote wound healing [189]. Inspired by this, exogenous electrical stimulation that mimics endogenous electric fields can effectively promote wound revascularization, reduce inflammation, promote tissue remodeling, and even induce wound re-epithelialization by regulating fibroblasts and keratinocytes [190,191]. Mao et al. [192] developed a novel porous hydrogel consisting of regenerated bacterial cellulose and MXene that had conductive properties to electrically stimulate cells. In in vitro experiments, the researchers demonstrated increased fibroblast proliferative activity after electrical stimulation (Fig. 19A). Moreover, the wound healing results in rats revealed that the wound area decreased, collagen deposition increased, angiogenesis increased, and growth factor expression was enhanced in the presence of exogenous electrical stimulation. The healing-promoting performance of electroactive hierarchical hydrogels was better than that of commercial dressings. However, the dressing of this system needs to be connected to an external power supply, which lacks convenience. Therefore, Fu et al. [193] proposed an electrically responsive hydrogel system combined with a wearable direct current (DC) pulsed piezoelectric nanogenerator, which could generate an electric current through the movement of the body to achieve continuous electrical stimulation therapy to promote wound healing (Fig. 19B). Wearable electronics featured with microneedles are also expected to be applied to wound electrotherapy [194,195].

## Summary and Prospect

With the in-depth understanding of wound healing and the progress of manufacturing technology, the development of wound dressings is no longer limited to a single protective barrier, and more and more functional hierarchical biomaterials have been proposed and applied to wound healing treatment. In the manuscript, we present the mechanisms of wound healing as well as key hindrances. In order to solve the problem of complex and refractory wound microenvironment and improve the therapeutic effect of wounds, hierarchical biomaterials with micro- and nano-porous structures or combined with nanomaterials have emerged. Their general functions and unique properties in wound applications of different organs are summarized. These hierarchical biomaterials provide new dressing options for promoting wound healing. However, they also face some challenges in practical application.

Firstly, hierarchical biomaterials from biological sources, such as gelatin, hyaluronic acid, and chitosan, have been reported and studied by numerous literatures in the past decades, and their good biocompatibility and even unique antibacterial property are conducive to practical applications. There are indeed some commercial products based on these materials that have entered clinical studies or even marketed, such as Tromboguard (components: alginate, chitosan, and polyurethane); Restore (components: sodium polyacrylate and hyaluronic acid); and DuoDerm (components: pectin, gelatin, carboxymethyl cellulose, and polyurethane) [196]. However, hierarchical biomaterials with nanoparticles for synergistic interaction require more in-depth long-term biosafety testing of nanomaterials. Secondly, more and more new and biogenic materials are still being proposed. For example, Chen et al. [197] found that membrane vesicles secreted by human symbiotic *L. reuteri* could regulate inflammation in wound tissue and promote the healing of mucous membranes and skin. Li et al. [198] proposed that microRNA-based strategies had potential value for the healing of corneal injury. These results show that more efficient wound dressings can still be expected in the future, where these new research results will be employed to fabricate novel hierarchical materials. Thirdly, current research in the field of skin wound healing focuses on improving wound healing rate, reducing inflammation, increasing angiogenesis and collagen deposition, and other functional repairs targeted at epithelial tissue. However, the natural skin barrier contains not only the epidermis and dermis but also skin appendages such as hair follicles and sebaceous glands, all of which work together. In addition, in recent years, organ-on-a-chip and organoid technologies have gradually matured, and it is also worth studying to combine skin-like tissues with hierarchical biomaterials to achieve complete skin functional repair. Last but not least, bioprinting technology has been shown to enable the establishment of biomimetic skin substitutes that contain skin cells to compensate for the lack of biocompatibility and functionality of traditional skin substitutes [199]. However, current biomimetic skin substitutes are still limited to the assembly of cells and scaffold materials, and regulatory strategies in graded biomaterials, such as Zn and Mg nanoparticles that promote cell proliferation, are expected to be combined with bioprinting technology to prepare more powerful biomimetic skin substitutes.

In general, although hierarchical biomaterials have made remarkable achievements in the field of wound treatment, they still have a long way to go. We hope that this review will help researchers understand the current development of hierarchical biomaterials and provide inspiration for the preparation of new and more efficient wound dressings.
